# K235 acetylation couples with PSPC1 to regulate the m^6^A demethylation activity of ALKBH5 and tumorigenesis

**DOI:** 10.1038/s41467-023-39414-4

**Published:** 2023-06-27

**Authors:** Xiao-Lan Zhang, Xin-Hui Chen, Binwu Xu, Min Chen, Song Zhu, Nan Meng, Ji-Zhong Wang, Huifang Zhu, De Chen, Jin-Bao Liu, Guang-Rong Yan

**Affiliations:** 1 Biomedicine Research Center, Guangdong Provincial Key Laboratory of Major Obstetric Disease, Guangdong Provincial Clinical Research Center for Obstetrics and Gynecology, the Third Affiliated Hospital of Guangzhou Medical University, Guangzhou, 510150 China; 2 Blood Transfusion Department, the Second Affiliated Hospital of Nanchang University, Nanchang, 330006 China; 3 The Sixth Affiliated Hospital of Guangzhou Medical University, Qingyuan People’s Hospital, Qingyuan, 511518 China; 4 Guangzhou Municipal and Guangdong Provincial Key Laboratory of Protein Modification and Degradation, State Key Laboratory of Respiratory Disease, School of Basic Medical Sciences, Guangzhou Medical University, Guangzhou, 511436 China

**Keywords:** RNA modification, Acetylation

## Abstract

N6-methyladenosine (m^6^A) modification plays important roles in bioprocesses and diseases. AlkB homolog 5 (ALKBH5) is one of two m^6^A demethylases. Here, we reveal that ALKBH5 is acetylated at lysine 235 (K235) by lysine acetyltransferase 8 and deacetylated by histone deacetylase 7. K235 acetylation strengthens the m^6^A demethylation activity of ALKBH5 by increasing its recognition of m^6^A on mRNA. RNA-binding protein paraspeckle component 1 (PSCP1) is a regulatory subunit of ALKBH5 and preferentially interacts with K235-acetylated ALKBH5 to recruit and facilitate the recognition of m^6^A mRNA by ALKBH5, thereby promoting m^6^A erasure. Mitogenic signals promote ALKBH5 K235 acetylation. K235 acetylation of ALKBH5 is upregulated in cancers and promotes tumorigenesis. Thus, our findings reveal that the m^6^A demethylation activity of ALKBH5 is orchestrated by its K235 acetylation and regulatory subunit PSPC1 and that K235 acetylation is necessary for the m^6^A demethylase activity and oncogenic roles of ALKBH5.

## Abstract (web summary)

Deregulation of N6-methyladenosine (m6A) modification can contribute to the pathogenesis of cancers. Here the authors show that m6A demethylase ALKBH5 is acetylated at K235 by acetyltransferase KAT8 and interacts with RNA-binding protein PSCP1 to enhance m6A demethylation and promote tumorigenesis.

## Introduction

N^6^-methyladenosine (m^6^A) is the most prevalent internal modification of eukaryotic mRNAs. In mammals, m^6^A is installed on mRNA by the m^6^A methyltransferases METTL3 and METTL14 (writers) and erased by the demethylases ALKBH5 and FTO (erasers)^1–5^. The m^6^A modification of mRNA plays critical roles in multiple fundamental cellular biological processes, including RNA splicing, stability, translation, sublocalization, and secondary structures^1,5–11^. Abnormal m^6^A on mRNA regulates the pathogenesis of multiple diseases, including cancer^1,12,13^.

ALKBH5 is one of two mammalian RNA m^6^A demethylases and can remove m^6^A methylation on mRNA^3,14^. ALKBH5 shares a nucleotide recognition lid and conserved active site residues with nucleic acid oxygenases such as FTO^15^. Knockout (KO) of ALKBH5 led to impaired spermatogenesis and male infertility in mice^3^. ALKBH5 expression can be induced by hypoxia and HIF1α in cancer cells^16^. Subsequent reports showed that ALKBH5 was upregulated and could facilitate cancer cell stemness and self-renewal, tumorigenesis, and progression by removing m^6^A from the mRNA of target genes in glioblastoma, acute myeloid leukemia, breast cancer, gastric cancer, and lung cancer; moreover, other studies have shown that ALKBH5 inhibits tumorigenesis and metastasis in lung cancer and pancreatic cancer^14,16–19^.

Paraspeckle component 1 (PSPC1) is an RNA-binding protein and represents one of three proteins found in paraspeckles, which are nuclear bodies located in the interchromatin space of the cell nucleus adjacent to speckles. Paraspeckles serve as dynamic molecular scaffolds for protein-protein and protein-nucleic acid interactions. PSPC1 is a paraspeckle marker that has been shown to participate in the regulation of transcription, RNA processing, RNA transport, RNA A-to-I editing, and DNA repair^20–22^.

In this study, we reveal that ALKBH5 is acetylated at K235 in response to mitogen stimulation and that K235 acetylation and deacetylation are mediated by the acetyltransferase KAT8 and the deacetylase HDAC7, respectively. K235 acetylation strengthens the m^6^A demethylation activity of ALKBH5 by increasing its binding to m^6^A on mRNA. RNA-binding protein PSPC1 is revealed here as a regulatory subunit of ALKBH5. PSPC1 binds to RNAs and preferentially interacts with K235-acetylated ALKBH5, recruiting and facilitating the recognition of m^6^A by ALKBH5, thereby promoting m^6^A removal from mRNA. Mitogenic signals stimulate ALKBH5 K235 acetylation, which promotes tumorigenesis. Thus, our study revealed that K235 acetylation of ALKBH5 couples with the ALKBH5 subunit PSPC1 to regulate the m^6^A demethylation activity of ALKBH5 and also revealed the crosstalk between protein acetylation and m^6^A modification of mRNA.

## Results

### ALKBH5 is acetylated at K235

**Fig. 1:**
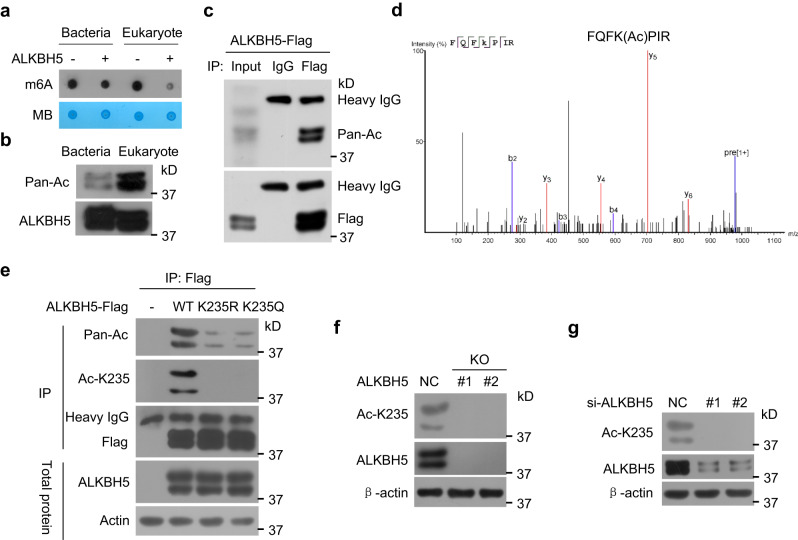
ALKBH5 is acetylated at K235. **a** Immunopurified ALKBH5 from HeLa cells or recombinant ALKBH5 from bacteria were incubated with m^6^A RNA oligos; the m^6^A levels were determined. **b** The acetylation levels of immunopurified ALKBH5 and recombinant ALKBH5 were determined with anti-pan acetylated lysine (Pan-Ac) antibody. **c** The ALKBH5-FLAG plasmid was transfected into HEK293T cells, ALKBH5-FLAG was immunoprecipitated (IPed) using anti-FLAG, and the acetylation of the IPed ALKBH5-FLAG was detected with anti-Pan-Ac antibody. **d** The K235 acetylation site was identified on IPed ALKBH5-FLAG using mass spectrometry. **e** The wild-type (WT) ALKBH5-FLAG and its mutant K235R and K235Q plasmids were transfected into HeLa cells, and the acetylation of the IPed WT and mutated ALKBH5-FLAG proteins was detected using pan acetylated lysine and anti-Ac K235 antibodies. **f** Endogenous K235 acetylation and ALKBH5 levels were detected in ALKBH5 KO HeLa cells. **g** HeLa cells were transfected with two anti-ALKBH5 siRNAs, and endogenous K235 acetylation and ALKBH5 levels were detected. Source data are provided as a Source Data file.

In the in vitro m^6^A-demethylation activity assays, surprisingly, we found that immunopurified ALKBH5 from eukaryotes had stronger demethylation activity and higher pan acetylation levels than recombinant ALKBH5 from bacteria (Fig. 1a, b). Therefore, the effects of ALKBH5 acetylation on the m^6^A demethylation activity of ALKBH5 are of interest. ALKBH5 was acetylated in cells when ALKBH5 was immunoprecipitated and detected with an anti-pan acetylated lysine antibody (Fig. 1c). A potential acetylated lysine site at 235 (K235) was further identified by mass spectrometry (Fig. 1d).

To confirm that ALKBH5 is acetylated at K235, ALKBH5 knockout (KO) cells were constructed using CRISPR‒Cas9 technology (Supplementary Fig. 1). We replaced K235 with either nonacetylated arginine (K235R) or acetyl-mimetic glutamine (K235Q) to construct ALKBH5 mutants. When wild-type ALKBH5 and its mutants K235R and K235Q were re-expressed in ALKBH5 KO cells, the acetylation of ALKBH5 was markedly reduced in the K235R and K235Q mutants compared with that of the wild-type ALKBH5 using the anti-pan acetylated lysine antibody (Fig. 1e), suggesting that most acetylation in ALKBH5 is K235 acetylation. We generated an antibody (Ac-K235) specific to K235-acetylated ALKBH5 and verified its specificity (Supplementary Fig. 2). K235-acetylated ALKBH5 was readily detected in wild-type ALKBH5 but not in the K235R and K235Q mutants (Fig. 1e). K235 acetylation was not detected in ALKBH5 KO cells (Fig. 1f). A similar result was obtained when ALKBH5 expression was knocked down using two anti-ALKBH5 siRNAs (Fig. 1g). Collectively, these results indicate that ALKBH5 is endogenously acetylated at K235 in cells.

### Acetyltransferase KAT8 acetylates ALKBH5 at K235

**Fig. 2:**
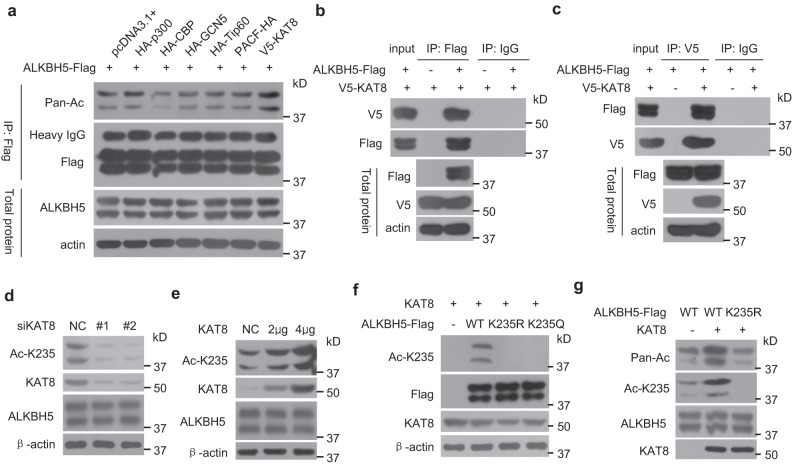
KAT8 is the acetyltransferase for ALKBH5 acetylation at K235. **a** KAT8 overexpression increased ALKBH5 acetylation at K235. HeLa cells were transfected with the indicated plasmids together with the ALKBH5-FLAG vector, ALKBH5-FLAG was IPed, and the acetylation of IPed ALKBH5-FLAG was determined using the anti-pan acetylated lysine antibody. **b**, **c** ALKBH5 interacted with KAT8. The ALKBH5-FLAG and KAT8-V5 plasmids were cotransfected into HeLa cells, ALKBH5-FLAG (**b**) and KAT8-V5 (**c**) complexes were co-IPed using anti-FLAG and anti-V5 antibodies, respectively, and KAT8-V5 and ALKBH5-FLAG were detected. **d** KD of KAT8 decreased the endogenous K235 acetylation of ALKBH5. HeLa cells were transfected with two anti-KAT8 siRNAs, and K235 acetylation was determined. **e** KAT8 overexpression increased the endogenous K235 acetylation of ALKBH5. HeLa cells were transfected with the indicated dose of the KAT8 plasmid, and K235 acetylation was determined. **f** The mutation of K235 eliminated the acetylation of KAT8 on ALKBH5 at K235. Wild-type ALKBH5 and its mutant K235R and K235Q plasmids were cotransfected with the KAT8 vector into ALKBH5 KO HeLa cells, and K235 acetylation was determined. **g** KAT8 directly acetylated ALKBH5, but not the mutant K235R, at K235 in the in vitro acetylation reaction. Immunopurified wild-type ALKBH5 and its mutant K235R were incubated with immunopurified KAT8, and ALKBH5 acetylation at K235 was determined using anti-Ac K235 and anti-pan acetylated lysine antibodies. Source data are provided as a Source Data file.

To identify the enzyme responsible for ALKBH5 K235 acetylation, we examined all six acetyltransferases: p300, CBP, GCN5, Tip60, PACF, and KAT8. We found that the ectopic expression of KAT8, but not the other five acetyltransferases, enhanced the K235 acetylation level of ALKBH5 (Fig. 2a). We further confirmed the interaction of KAT8 with ALKBH5 (Fig. 2b, c) and showed that ALKBH5 did not interact with other acetyltransferases such as CBP and Tip60 (Supplementary Fig. 3). Knockdown (KD) of KAT8 markedly decreased the K235 acetylation of endogenous ALKBH5 (Fig. 2d), whereas overexpression of KAT8 increased the K235 acetylation of endogenous ALKBH5 in a dose-dependent manner (Fig. 2e). KAT8 did not induce the K235 acetylation of ALKBH5 when the K235 site was mutated to K235R or K235Q (Fig. 2f). Furthermore, immunopurified KAT8 directly and efficiently increased the pan acetylation and K235 acetylation levels of immunopurified wild-type ALKBH5 in an in vitro acetylation assay (Fig. 2g); however, this activity was not observed in the K235R mutant. Together, these results indicate that KAT8 is the acetyltransferase of ALKBH5 at K235.

### Deacetylase HDAC7 deacetylates ALKBH5 at K235

**Fig. 3:**
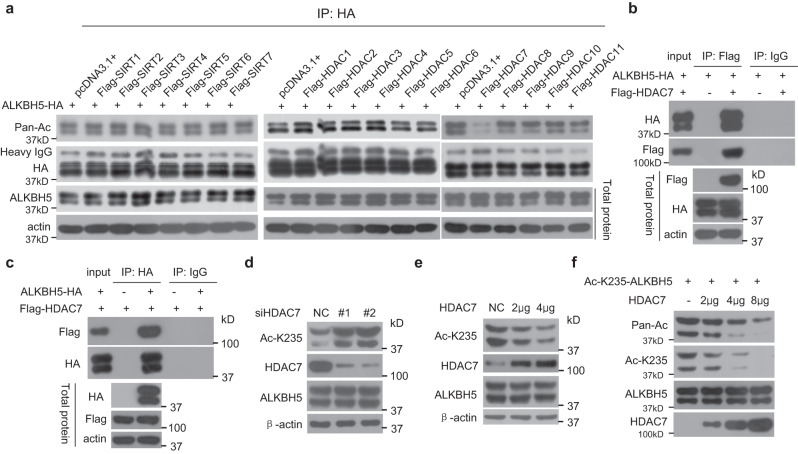
HADC7 is the deacetylase for ALKBH5 acetylation at K235. **a** HADC7 overexpression reduced ALKBH5 acetylation at K235. HeLa cells were transfected with the indicated plasmids together with the ALKBH5-HA vector; ALKBH5-HA was IPed using anti-HA antibody, and K235 acetylation in the IPed ALKBH5-HA was determined using the anti-pan acetylated lysine antibody. **b**, **c** ALKBH5 interacted with HDAC7. The ALKBH5-HA and HDAC7-FLAG plasmids were cotransfected into HeLa cells, and the HDAC7-FLAG (**b**) and ALKBH5-HA (**c**) complexes were co-IPed using anti-FLAG and anti-HA antibodies, respectively. ALKBH5-HA and HDAC7-FLAG were detected. **d** Silencing of HDAC7 increased the endogenous K235 acetylation of ALKBH5. HeLa cells were transfected with anti-HDAC7 siRNAs, and K235 acetylation was determined. **e** HDAC7 overexpression decreased the endogenous K235 acetylation of ALKBH5. HeLa cells were transfected with the indicated dose of HDAC7 plasmid, and K235 acetylation was determined. **f** HDAC7 directly deacetylated ALKBH5 at K235 in a dose-dependent manner in the in vitro deacetylation reaction. Immunopurified wild-type ALKBH5 containing K235-acetylated ALKBH5 stimulated by KAT8 overexpression was incubated with the indicated dose of immunopurified HADC7, and ALKBH5 acetylation at K235 was determined using anti-Ac K235 and anti-pan acetylated lysine antibodies. Source data are provided as a Source Data file.

Eighteen mammalian histone deacetylases (HDACs) have been identified, including class I-II HDAC1-11 and class III SIRT1-7^23^. To identify the enzyme responsible for ALKBH5 K235 deacetylation, we examined all eighteen HDACs/SIRTs. We found that the ectopic expression of HDAC7, but not the other seventeen deacetylases, markedly reduced the acetylation level of ALKBH5 (Fig. 3a). The interaction of HADC7 with ALKBH5 was further confirmed (Fig. 3b, c). KD of HDAC7 markedly enhanced the K235 acetylation level of endogenous ALKBH5 (Fig. 3d), whereas ectopic expression of HDAC7 decreased the K235 acetylation level of endogenous ALKBH5 in a dose-dependent manner (Fig. 3e). Furthermore, K235-acetylated ALKBH5 was immunopurified from cells in which KAT8 was ectopically overexpressed. The immunopurified HDAC7 directly and efficiently reduced the pan acetylation and K235 acetylation of the immunopurified K235-acetylated ALKBH5 in a dose-dependent manner in an in vitro deacetylation assay (Fig. 3f). Collectively, these results indicate that HADC7 is the deacetylase of ALKBH5 at K235.

### K235 acetylation of ALKBH5 is critical for its RNA m^6^A demethylation activity

**Fig. 4:**
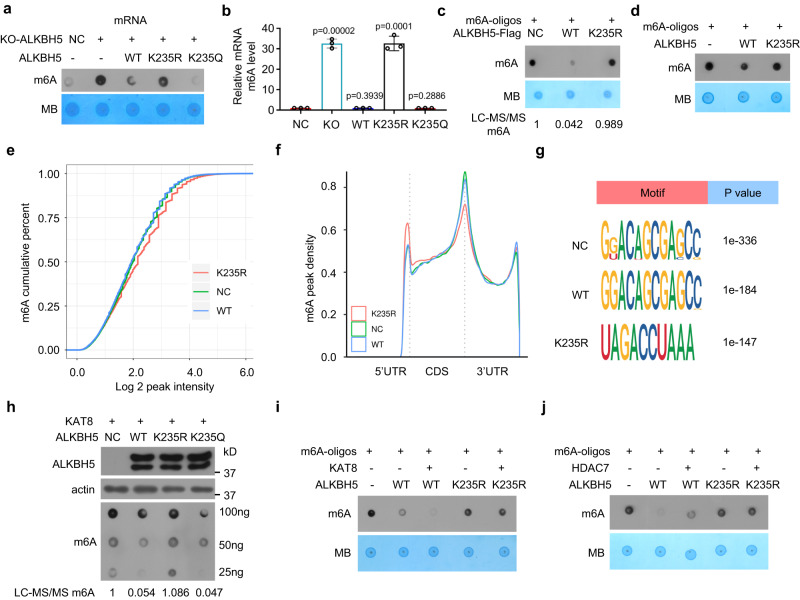
ALKBH5 acetylation at K235 is critical for the RNA m^6^A demethylation activity of ALKBH5. **a**, **b** K235 acetylation of ALKBH5 decreased the cellular mRNA m^6^A levels. The wild-type ALKBH5 and its mutant K235R and K235Q plasmids were transfected into ALKBH5 KO HeLa cells, and the cellular mRNA m^6^A level was determined by dot blotting (**a**) and quantified by LC‒MS/MS analysis (**b**) (n = 3, two-tailed unpaired Student’s t test, mean ± SD). **c**, **d** Wild-type ALKBH5, but not the K235R mutant, directly demethylated m^6^A in the m^6^A-RNA oligos in the in vitro demethylation reaction. Immunopurified wild-type ALKBH5 and its mutant K235R (**c**) or recombinant wild-type ALKBH5 and its mutant K235R (**d**) were incubated with m^6^A RNA oligos; the m^6^A level was determined by dot blotting or LC‒MS/MS assays. **e** Cumulative distribution curve for the m^6^A peak abundance in NC, WT and K235R cells. **f** Distribution of m^6^A peaks in the 5′ UTR, CDS, stop codon and 3′ UTR in NC, WT and K235R cells. **g** Top consensus motif identified by HOMER with m^6^A peaks in NC, WT and K235R cells. **h** The indicated ALKBH5 vectors together with the KAT8 plasmid were cotransfected into ALKBH5 KO HeLa cells, and the cellular RNA m^6^A level was determined. **i**, **j** Recombinant wild-type ALKBH5 or its K235R mutant was incubated with m^6^A RNA oligos after recombinant wild-type ALKBH5 or its K235R mutant was treated with immunopurified KAT8 (**i**) or immunopurified HDAC7 (**j**), and the m^6^A level was determined. Source data are provided as a Source Data file.

To determine whether K235 acetylation of ALKBH5 affects the RNA m^6^A demethylation activity of ALKBH5, wild-type ALKBH5 or its mutants K235R or K235Q were re-expressed in ALKBH5 KO cells, and the cellular m^6^A levels in total RNA and mRNA were assayed. The re-expression of wild-type ALKBH5 and acetyl-mimetic glutamine mutant K235Q reduced the RNA m^6^A level to that in the negative control (NC) cells, whereas the K235R mutant did not reduce the RNA m^6^A level (Fig. 4a, b, Supplementary Figs. 4 and 5a). Similar results were obtained when wild-type ALKBH5 and K235 mutants, which were resistant to anti-ALKBH5 siRNA, were re-expressed in HCT-116 cells in which ALKBH5 expression was knocked down by anti-ALKBH5 siRNA (Supplementary Fig. 5b). Furthermore, we purified wild-type ALKBH5 and K235R mutants and measured the m^6^A demethylation activity of ALKBH5 in an in vitro demethylation assay and found that the immunopurified wild-type ALKBH5 complexes could directly and efficiently decrease the RNA m^6^A level of synthetic m^6^A-modified oligonucleotide substrates, whereas the K235R mutant could not decrease the RNA m^6^A level (Fig. 4c). We expressed and purified the recombinant ALKBH5 protein from bacteria, and recombinant ALKBH5 was also K235-acetylated (Fig. 1b, Line 3 and Lane 4 in Fig. 6g), similar to some proteins in bacteria^24,25^. Recombinant wild-type ALKBH5 also directly and efficiently decreased the m^6^A level of m^6^A-RNA oligos in the in vitro demethylation reaction, whereas the recombinant K235R mutant did not decrease the m^6^A level (Fig. 4d).

Next, we performed transcriptome m^6^A sequencing to compare the m^6^A profiles of the negative control (NC) cells and ALKBH5 KO cells stably re-expressing wild-type ALKBH5 (WT) or its mutant K235R (K235R). In total, m^6^A-seq identified 33044, 33561, and 34447 m^6^A peaks in NC, WT, and K235R cells, respectively. Compared with NC cells, 7011 m^6^A peaks, including 2247 upregulated peaks, were significantly altered in ALKBH5 KO cells stably re-expressing wild-type ALKBH5 (WT) (p < 0.05); 10617 m^6^A peaks, including 6703 upregulated peaks, were significantly altered in ALKBH5 KO cells stably re-expressing the ALKBH5 K235R mutant (K235R). Compared with WT cells, 10648 m^6^A peaks, including 7931 upregulated peaks, were altered in K235R cells. More m^6^A peaks, particularly the upregulated m^6^A peaks, were altered in K235R cells than in WT cells compared with NC cells. The overall cellular RNA m^6^A abundance in WT cells was restored to that in NC cells, whereas the K235R cells did not exhibit restoration of the cellular RNA m^6^A abundance (Fig. 4e). The distribution of the m^6^A peaks in the 5′ UTR, coding DNA sequence (CDS), stop codon and 3′ UTR between the NC and WT cells was very similar but differed from that in the K235R cells (Fig. 4f). The most common m^6^A motif “GGACA” was significantly enriched in m^6^A peaks in the NC and WT cells, and the m^6^A motif was highly similar between the NC and WT cells but different from that in the K235R cells (Fig. 4g). Therefore, as expected, the disappearance of ALKBH5 K235 acetylation eliminated RNA m^6^A demethylase activity and increased RNA m^6^A abundance, whereas the re-expression of wild-type ALKBH5, but not the K235R mutant, recovered the alteration of m^6^A induced by ALKBH5 KO.

Furthermore, the overexpression of the acetyltransferase KAT8 of ALKBH5 decreased the cellular RNA m^6^A, whereas the KD of KAT8 increased the cellular RNA m^6^A (Supplementary Fig. 5c, d). Overexpression of the deacetylase HADC7 of ALKBH5 enhanced cellular RNA m^6^A, whereas KD of HDAC7 reduced cellular mRNA m^6^A (Supplementary Fig. 5e, f). KAT8 overexpression promoted the reduction of cellular RNA m^6^A induced by wild-type ALKBH5 and K235Q re-expression in ALKBH5 KO cells but not by K235R re-expression (Fig. 4h, Supplementary Fig. 5g). Increasing K235 acetylation of recombinant ALKBH5, which was induced by purified KAT8, increased the removal of m^6^A on m^6^A-RNA oligos in the in vitro demethylation reaction, but the recombinant K235R mutant did not show the same effect (Fig. 4i). Decreasing K235 acetylation of recombinant ALKBH5, which was induced by purified HDAC7, impaired the removal of m^6^A from m^6^A-RNA oligos, but the recombinant K235R mutant did not show the same effect (Fig. 4j). Immunopurified wild-type ALKBH5 from KAT8-overexpressing cells and HDAC7 KD cells, but not the K235R mutant, directly and efficiently removed m^6^A from m^6^A-RNA oligos (Supplementary Fig. 5h, k), whereas immunopurified wild-type ALKBH5 from KAT8 KD cells and HDAC7-overexpressing cells, but not the K235R mutant, did not directly and efficiently remove m^6^A from m^6^A-RNA oligos (Supplementary Fig. 5i, j). Taken together, ALKBH5 K235 acetylation is critical for the RNA m^6^A demethylation activity of ALKBH5.

### K235 acetylation increases the recognition of RNA m^6^A by ALKBH5

**Fig. 5:**
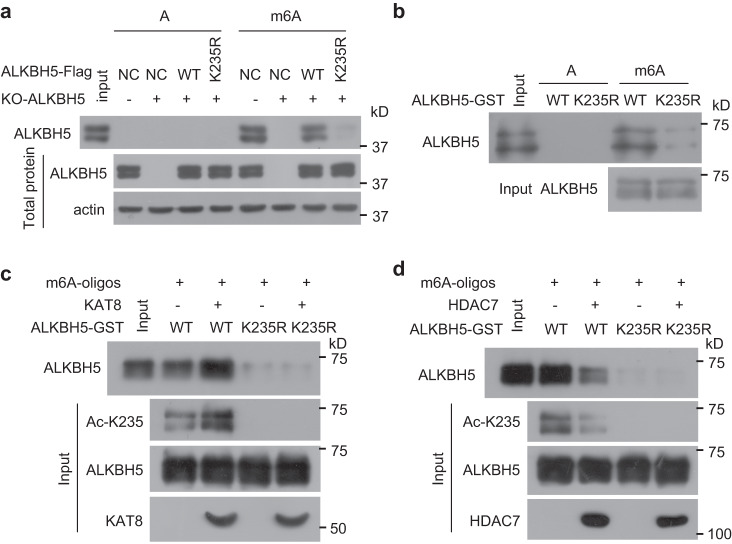
K235 acetylation of ALKBH5 increased its binding to RNA m^6^A. **a** The in vitro binding of wild-type ALKBH5 and its mutant K235R to m^6^A-unmethylated or methylated RNA oligos was investigated in ALKBH5 KO HeLa cells stably re-expressing wild-type ALKBH5 or its mutant K235R using RNA pulldown assays. **b** The in vitro binding of recombinant wild-type ALKBH5 and its mutant K235R to m^6^A-unmethylated or methylated RNA oligos was investigated. **c**, **d** The in vitro binding of recombinant wild-type ALKBH5 and its mutant K235R to m^6^A-unmethylated or methylated RNA oligos was investigated after the recombinant wild-type ALKBH5 or its mutant K235R were treated by immunopurified KAT8 (**c**) or by immunopurified HDAC7 (**d**). Source data are provided as a Source Data file.

To investigate how K235 acetylation affects the m^6^A demethylation activity of ALKBH5, the influences of K235 acetylation on ALKBH5 stability, subcellular localization, and RNA m^6^A recognition were determined. We found that the absence of K235 acetylation impaired the recognition and binding of ALKBH5 to RNA m^6^A (Fig. 5a) but did not alter the protein level, half-life or sublocalization of ALKBH5 (Supplementary Fig. 6a–d). Relative to recombinant wild-type ALKBH5, the binding of the recombinant K235R mutant to RNA m^6^A was markedly impaired (Fig. 5b). Increasing K235 acetylation of recombinant ALKBH5, which was induced by purified KAT8, enhanced the binding of recombinant ALKBH5 to RNA m^6^A, but not that of the recombinant K235R mutant (Fig. 5c). Decreasing K235 acetylation of recombinant ALKBH5, which was induced by purified HDAC7, impaired the binding of recombinant ALKBH5 to RNA m^6^A, but not that of the recombinant K235R mutant (Fig. 5d). Therefore, K235 acetylation of ALKBH5 promotes the binding and recognition of RNA m^6^A by ALKBH5.

### K235-acetylated ALKBH5 preferentially binds to the RNA-binding protein PSPC1

**Fig. 6:**
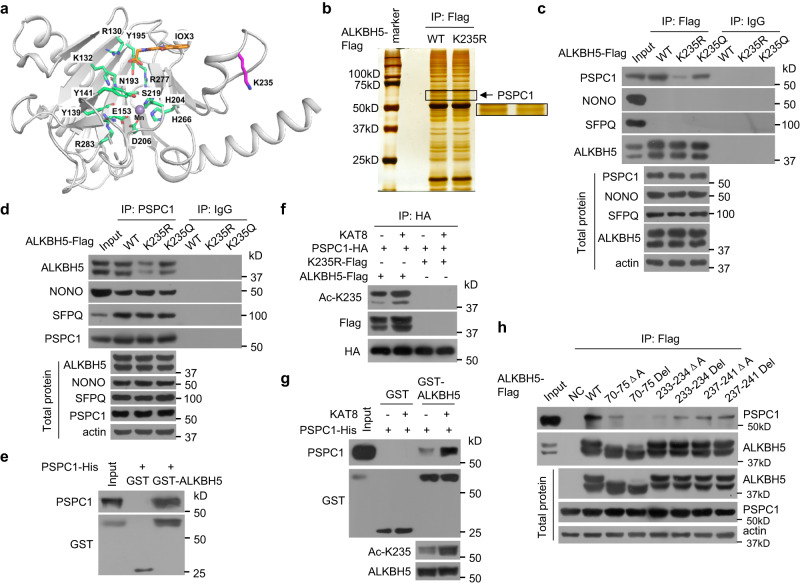
The RNA-binding protein PSPC1 preferentially interacts with K235-acetylated ALKBH5. **a** K235 is distant from the active site of ALKBH5. The relative position of K235 (magenta) with respect to the active site, is depicted by a collection of functionally important residues shown as green sticks. The covalent inhibitor IOX3 is shown in orange, and the active site manganese is shown as a sphere. The structure was drawn using the structure PBD 4NJ4. **b** PSPC1 was identified to preferentially interact with ALKBH5, and not its K235R mutant, based on the interactomics assay. The wild-type ALKBH5 or its mutant K235R plasmids together with the KAT8 vector were cotransfected into ALKBH5 KO HeLa cells, and the co-IPed wild-type ALKBH5 and its mutant K235R complexes were identified by mass spectrometry. **c**, **d** ALKBH5-FLAG (**c**) and PSPC1 (**d**) complexes were IPed in ALKBH5 KO HeLa cells stably re-expressing wild-type ALKBH5-FLAG or its mutants K235R or K235Q using anti-FLAG and PSPC1 antibodies, respectively, and the indicated proteins were detected. **e** The direct binding of recombinant GST-ALKBH5 to recombinant PSPC1-His was analyzed using a GST pull-down assay. **f** The PSPC1-HA plasmid together with the KAT8 plasmid were cotransfected into ALKBH5 KO HeLa cells stably re-expressing wild-type ALKBH5-FLAG or the K235R mutant. The PSPC1-HA complexes were IPed using anti-HA, and K235 acetylation and ALKBH5-FLAG were detected. **g** Recombinant GST-ALKBH5 was K235-acetylated by immunopurified KAT8, and the direct binding of recombinant K235-acetylated GST-ALKBH5 to recombinant PSPC1-His was analyzed using a GST pull-down assay. **h** The indicated ALKBH5-FLAG mutants were transfected into ALKBH5 KO HeLa cells, ALKBH5-FLAG complexes were IPed using an anti-FLAG antibody, and PSPC1 was detected. A total of 70–75ΔA and 70–75 Del indicate that aa 70–75 in ALKBH5 were mutated into AAAAAA and deleted, respectively. Source data are provided as a Source Data file.

As shown in Fig. 6a, based on an analysis of the ALKBH5 structure (PDB: 4NJ4)^15^, the side chain of K235 is located on a loop that is distal to the structurally compact active site of ALKBH5, and K235 acetylation modification is on a surface that is accessible to binding partners, suggesting that K235 acetylation is unlikely to affect the catalytic activity of ALKBH5. We speculate that K235 acetylation may affect the binding of ALKBH5 to its regulatory subunits, thereby modulating the binding and recognition of RNA m^6^A by ALKBH5 and the removal of m^6^A in RNAs. However, to date, no regulatory subunits of ALKBH5 for erasing RNA m^6^A have been reported. Therefore, we further investigated the regulatory subunits of ALKBH5 for erasure of RNA m^6^A. The interactors of wild-type ALKBH5 and K235R mutants were investigated by coimmunoprecipitation and mass spectrometry analyses in which the acetyltransferase KAT8 of ALKBH5 was overexpressed in cells. Interactomics analysis showed that RNA-binding protein PSPC1 referentially interacts with wild-type ALKBH5 relative to its K235R mutant (Fig. 6b), suggesting that PSPC1 may be a potential regulatory subunit of ALKBH5 for erasing RNA m^6^A.

We further investigated the influence of K235 acetylation on the interaction of ALKBH5 with PSPC1. We found that K235 deacetylation impaired the binding of ALKBH5 to endogenous PSPC1 and the colocalization of ALKBH5 with endogenous PSPC1 under normal cell culture conditions, whereas the acetyl-mimetic glutamine mutant K235Q retained the ability to interact and colocalize with PSPC1 (Fig. 6c, d, Supplementary Fig. 6d). Previous studies have shown that PSPC1 forms dimers and polymers with other RNA-binding proteins such as NONO and SFPQ^20^. We found that endogenous NONO and SFPQ did not bind to wild-type ALKBH5 or its mutants K235R and K235Q (Fig. 6c), which is consistent with the interactome data (Fig. 6b), whereas NONO and SFPQ interacted with PSPC1 as previously reported (Fig. 6d), suggesting that PSPC1 formed other complexes with K235-acetylated ALKBH5. GST pull-down assays further showed that recombinant ALKBH5 directly bound to recombinant PSPC1 (Fig. 6e). Increasing the ALKBH5 K235 acetylation level, which was induced by the overexpression of KAT8, strengthened the interaction of ALKBH5 with PSPC1 but not the interaction of the ALKBH5 K235R mutant with PSPC1 (Fig. 6f). Increasing K235 acetylation of recombinant ALKBH5, which was induced by purified KAT8, increased the direct binding of recombinant ALKBH5 to recombinant PSPC1 (Fig. 6g). Collectively, these results indicate that K235 acetylation is essential for the interaction of ALKBH5 with PSPC1.

We speculate that the helix near K235 may be required for PSPC1 binding. The deletion of 70-75 aa (70-75 Del) near K235 in the ALKBH5 helix eliminated the interaction of ALKBH5 with PSPC1 (Fig. 6h), indicating that the 70-75 aa region near K235 in the ALKBH5 helix is required for the binding of ALKBH5 to PSPC1.

ALKBH5 has been shown to colocalize with mRNA-processing factors such as phosphorylated SC35 (SC35-pi) in nuclear speckles, and ALKBH5 KD resulted in the disappearance of SC35-pi staining^3^. Wild-type ALKBH5 and K235Q mutant re-expression restored the signals of SC35-pi staining in ALKBH5 KO cells, whereas the re-expression of the ALKBH5 K235R mutant failed to do so for the effects induced by ALKBH5 KD (Supplementary Fig. 6e); this is similar to the previously reported function of the demethylation-inactive mutant ALKBH5 H204A in which the iron ligand residue H204 was substituted with Ala^3^. The wild-type ALKBH5 and K235Q mutant colocalized with the nuclear speckle marker SC35-pi (Supplementary Fig. 6e), and PSPC1 also colocalized with SC35-pi under the condition of wild-type ALKBH5 or K235Q mutant expression (Supplementary Fig. 6f), suggesting that K235-acetylated ALKBH5 and PSPC1 were colocalized in nuclear speckles. Collectively, these results indicate that K235 acetylation promotes the interaction of ALKBH5 with PSPC1.

### PSPC1 is a regulatory subunit of ALKBH5 required for erasing RNA m^6^A

**Fig. 7:**
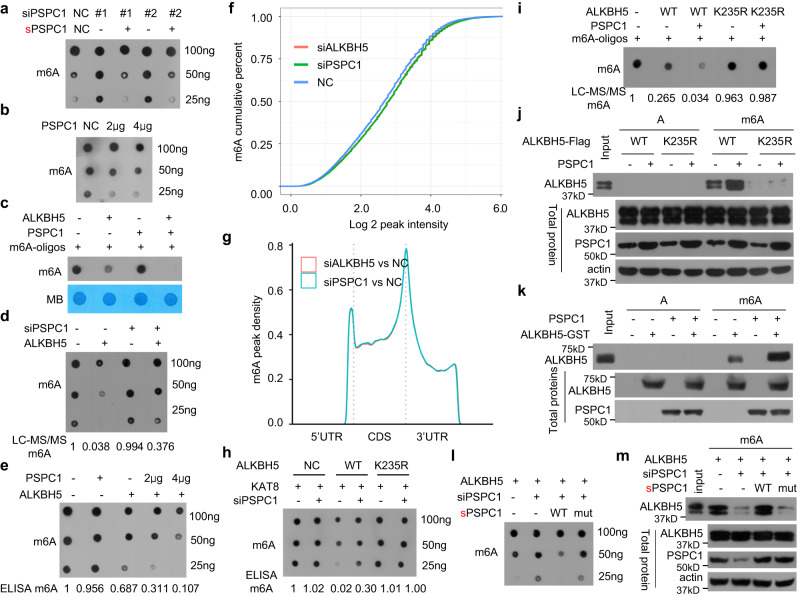
PSPC1 is a regulatory subunit of ALKBH5 and preferentially interacts with K235-acetylated ALKBH5 to recruit and facilitate the recognition of RNA m^6^A by ALKBH5, thereby promoting RNA m^6^A removal by ALKBH5. **a** PSPC1 KD increased cellular RNA m^6^A levels, whereas PSPC1 re-expression decreased cellular RNA m^6^A levels in PSPC1 KD cells. **b** HeLa cells were transfected with the indicated dose of PSPC1 plasmid, and cellular RNA m^6^A was determined. **c** Recombinant ALKBH5 was incubated with recombinant PSPC1 and m^6^A RNA oligos, and the m^6^A level was determined. **d** ALKBH5 plasmid together with anti-PSPC1 siRNA were cotransfected into ALKBH5 KO HeLa cells, and cellular RNA m^6^A was determined by dot blotting and LC‒MS/MS. **e** ALKBH5 plasmid together with PSPC1 plasmid were cotransfected into ALKBH5 KO HeLa cells, and cellular RNA m^6^A was determined by dot blotting and m^6^A ELISA. **f** Cumulative distribution curve for the abundance in NC, ALKBH5 KD and PSPC1 KD cells. **g** Distribution of m^6^A peaks in the 5′ UTR, CDS, stop codon and 3′ UTR between ALKBH5 KD-mediated m^6^A peaks and PSPC1 KD-mediated m^6^A peaks. **h** KAT8 plasmid together with anti-PSPC1 siRNA were cotransfected into ALKBH5 KO HeLa cells stably re-expressing the wild-type ALKBH5-FLAG or K235R mutant, and the cellular RNA m^6^A level was determined. **i** Immunopurified wild-type ALKBH5 or its K235R mutant was incubated with immunopurified PSPC1 and m^6^A RNA oligos, and the m^6^A level was determined. **j** The PSPC1 plasmid was transfected into ALKBH5 KO HeLa cells stably re-expressing wild-type ALKBH5-FLAG or K235R mutant, and the in vitro binding of wild-type ALKBH5 and K235R mutant to m^6^A-unmethylated or methylated RNA oligos was investigated. **k** The in vitro direct binding of recombinant ALKBH5 to m^6^A-unmethylated or methylated RNA oligos was investigated. **l**, **m** Anti-PSPC1 siRNAs with the wild-type sPSPC1 plasmid or the sPSPC1 mutant, which does not bind to RNAs, were cotransfected into ALKBH5 KO HeLa cells stably re-expressing wild-type ALKBH5-FLAG, and cellular RNA m^6^A was determined (**l**). The in vitro binding of ALKBH5 to m^6^A-methylated RNA oligos was investigated (**m**). Source data are provided as a Source Data file.

We investigated the effect of PSPC1 on ALKBH5-mediated RNA m^6^A removal. The KD of PSPC1 increased the cellular RNA m^6^A levels, whereas PSPC1 re-expression decreased the cellular RNA m^6^A levels in PSPC1 KD cells (Fig. 7a, Supplementary Fig. 7a). The overexpression of PSPC1 decreased the cellular RNA m^6^A levels (Fig. 7b, Supplementary Fig. 7b). Furthermore, we found that neither recombinant PSPC1 itself nor purified PSPC1-FLAG from ALKBH5 KO cells could directly and efficiently decrease the RNA m^6^A levels of synthetic m^6^A-modified oligonucleotide substrates (Lane 3 in Fig. 7c, Supplementary Fig. 7c), suggesting that PSPC1 itself is not a demethylase for RNA m^6^A. The erasing of cellular RNA m^6^A by ALKBH5 was markedly blocked when the expression of PSPC1 was silenced in cells (Fig. 7d, Supplementary Fig. 7d), whereas PSPC1 overexpression increased the erasing of cellular RNA m^6^A by ALKBH5 in a dose-dependent manner (Fig. 7e, Supplementary Fig. 7e). As expected, recombinant ALKBH5 directly removed the RNA m^6^A modification on m^6^A RNA oligos, and recombinant PSPC1 further directly increased the removal of the m^6^A modification on m^6^A RNA oligos by recombinant ALKBH5 in the in vitro demethylation reaction (Fig. 7c). The immunopurified PSPC1 also directly and efficiently increased the erasing effects of immunopurified ALKBH5 on RNA m^6^A from synthetic m^6^A-modified oligonucleotide substrates in the in vitro demethylation reaction (Lane 3 in Fig. 7i).

We next performed transcriptome m^6^A sequencing to compare the RNA m^6^A targets regulated by ALKBH5 and PSPC1. A total of 5856 and 6111 m^6^A peaks were altered by ALKBH5 KD and PSPC1 KD, respectively. Among them, approximately 58% of the m^6^A peaks were commonly regulated by ALKBH5 KD and PSPC1 KD. As shown in Fig. 7f, both ALKBH5 KD and PSPC1 KD increased the overall cellular RNA m^6^A abundance, and the trends were highly similar. The distributions of the ALKBH5-regulated m^6^A peaks and the PSPC1-regulated m^6^A peaks in the 5′ UTR, CDS, stop codon, and 3′ UTR were highly similar (Fig. 7g). These results indicate that the m^6^A targets mediated by ALKBH5 were highly similar to those mediated by PSPC1. Together, we show that PSPC1 is a regulatory subunit of ALKBH5 required for erasing RNA m^6^A.

### ALKBH5 K235 acetylation is critical for the effects of PSPC1 on demethylation activity of ALKBH5 by facilitating the binding of ALKBH5 to RNA m^6^A

Next, we demonstrated that the absence of K235 acetylation eliminated the synergistic effects of ALKBH5 and KD of PSPC1 on removing cellular RNA m^6^A (Fig. 7h). The immunopurified PSPC1 directly and efficiently increased the erasing effects of immunopurified ALKBH5 on RNA m^6^A from synthetic m^6^A-modified oligonucleotide substrates in the in vitro demethylation reaction, but not the erasing effects of ALKBH5 mutant K235R (Fig. 7i). Taken together, K235 acetylation of ALKBH5 is critical for the effects of PSPC1 on the demethylation of ALKBH5.

We further showed that PSPC1 overexpression increased the binding and recognition of RNA m^6^A by ALKBH5 (Lanes 6 and 7 in Fig. 7j), whereas PSPC1 KD destroyed the binding and recognition of RNA m^6^A by ALKBH5 (Lanes 6 and 7 in Supplementary Fig. 7g) but did not change the protein level or sublocalization of the RNA m^6^A demethylases ALKBH5 and FTO (Supplementary Fig. 8). Recombinant PSPC1 increased the direct binding of recombinant ALKBH5 to RNA m^6^A (Fig. 7k). A previous study showed that PSPC1 bearing four mutations in its RNA binding domains (F118A/F120A/K197A/F199A, hereafter PSPC1mut) does not bind to RNAs^26^. PSPC1 did not increase the erasing of cellular RNA m^6^A by ALKBH5 or the binding of ALKBH5 to RNA m^6^A when the binding of PSPC1 to RNAs was eliminated (Fig. 7l, m, Supplementary Fig. 7h), implying that the binding of PSPC1 to RNA is essential for the effects of PSPC1 on the demethylation activity of ALKBH5 and the binding of ALKBH5 to RNA m^6^A.

Furthermore, PSPC1 overexpression increased the recognition of RNA m6A by wild-type ALKBH5 but not by the ALKBH5 K235R mutant (Fig. 7j), whereas PSPC1 KD destroyed the binding of wild-type ALKBH5 but not that of the K235R mutant to RNA m^6^A (Supplementary Fig. 7g), suggesting that K235 acetylation of ALKBH5 modulates the stimulatory effect of PSPC1 on the binding and recognition of RNA m^6^A by ALKBH5 Taken together, K235 acetylation of ALKBH5 is critical for the effect of PSPC1 on the m^6^A demethylation of ALKBH5 by facilitating the recognition of RNA m^6^A by ALKBH5.

### Mitogenic signals stimulate K235 acetylation of ALKBH5 and the interaction of ALKBH5 with PSPC1 to reduce RNA m^6^A levels

**Fig. 8:**
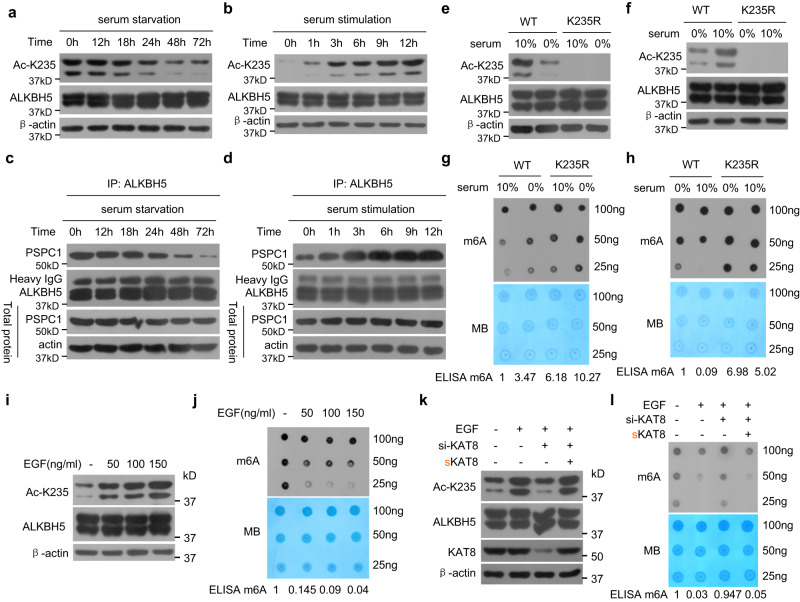
Mitogenic signals stimulate ALKBH5 acetylation at K235, increase the interaction of ALKBH5 with PSPC1 and decrease cellular RNA m^6^A. **a**, **c** Serum deprivation decreased K235 acetylation of ALKBH5 and the interaction of ALKBH5 with PSPC1. HeLa cells were serum starved (0% FBS) for different times, K235 acetylation was determined (**a**), and the interaction of ALKBH5 with PSPC1 was analyzed (**c**). **b**, **d** Serum stimulation increased ALKBH5 K235 acetylation and the interaction of ALKBH5 with PSPC1. HeLa cells were serum stimulated with the addition of 10% FBS after 2 days of serum deprivation, K235 acetylation was determined (**b**), and the interaction of ALKBH5 with PSPC1 was analyzed (**d**). **e**, **g** K235 deacetylation eliminated the reduction in ALKBH5 K235 acetylation and RNA m^6^A enhancement induced by serum deprivation. ALKBH5 KO HeLa cells stably re-expressing wild-type ALKBH5 or the K235R mutant were serum starved for 72 h, K235 acetylation was determined (**e**), and the cellular RNA m^6^A level was analyzed (**g**). **f**, **h** K235 deacetylation eliminated the enhancement of ALKBH5 K235 acetylation and the reduction in RNA m^6^A induced by serum stimulation. ALKBH5 KO HeLa cells stably re-expressing wild-type ALKBH5 or K235R mutant were serum stimulated with the addition of 10% FBS for 12 h after 2 days of serum deprivation, K235 acetylation was determined (**f**), and the cellular RNA m^6^A level was analyzed (**h**). **i**, **j** EGF increased ALKBH5 K235 acetylation and reduced cellular RNA m^6^A levels. HeLa cells were treated with different doses of EGF, K235 acetylation was determined (**i**), and the cellular RNA m^6^A level was analyzed (**j**). **k**, **l** EGF increased ALKBH5 K235 acetylation and reduced cellular RNA m^6^A levels through KAT8. HeLa cells were treated with EGF for 24 h after cotransfection with anti-KAT8 siRNA and synonymously mutated sKAT8 plasmid, which was resistant to anti-KAT8 siRNA; K235 acetylation was determined (**k**), and the cellular RNA m^6^A level was analyzed (**l**). Source data are provided as a Source Data file.

Next, we investigated which signals promote ALKBH5 K235 acetylation. We first examined the ALKBH5 K235 acetylation levels in response to serum deprivation and stimulation. Serum deprivation dramatically decreased the cellular ALKBH5 K235 acetylation level in cancer cells within 24 h but did not alter the ALKBH5 level (Fig. 8a). Conversely, the addition of serum to serum-starved cancer cells enhanced the ALKBH5 K235 acetylation level within 3 h (Fig. 8b). Serum deprivation reduced the interaction of ALKBH5 and PSPC1 (Fig. 8c), whereas serum restimulation of serum-starved cells enhanced the interaction of ALKBH5 with PSPC1 (Fig. 8d). Serum deprivation increased the mRNA m^6^A level, whereas serum restimulation of serum-starved cells decreased the mRNA m^6^A level (Supplementary Fig. 9). However, serum deprivation and restimulation did not change the K235 acetylation of ALKBH5 when the K235 site was mutated to K235R in ALKBH5 (Fig. 8e, f). Serum deprivation and restimulation did not affect the cellular RNA m^6^A level when the K235 site was mutated in ALKBH5 (Fig. 8g, h).

Furthermore, we found that the mitogenic factor EGF increased K235 acetylation of ALKBH5 in a dose-dependent manner but did not alter ALKBH5 protein levels (Fig. 8i) and reduced cellular RNA m^6^A (Fig. 8j). To confirm that EGF increased ALKBH5 K235 acetylation through the acetyltransferase KAT8 of ALKBH5, KAT8 expression was knocked down during EGF treatment. KD of KAT8 completely blocked the enhancement of ALKBH5 K235 acetylation induced by EGF treatment, whereas KAT8 re-expression in KAT8 KD cells restored the enhancement of ALKBH5 K235 acetylation induced by EGF treatment (Fig. 8k). KAT8 KD attenuated the reduction in cellular RNA m^6^A induced by EGF treatment, whereas KAT8 re-expression recovered the reduction in cellular RNA m^6^A levels induced by EGF treatment (Fig. 8l). Collectively, our results indicate that mitogenic signals promote ALKBH5 K235 acetylation and ALKBH5 binding to PSPC1 and decrease RNA m^6^A through KAT8.

### K235 acetylation of ALKBH5 is critical for the roles of ALKBH5 in tumorigenesis

**Fig. 9:**
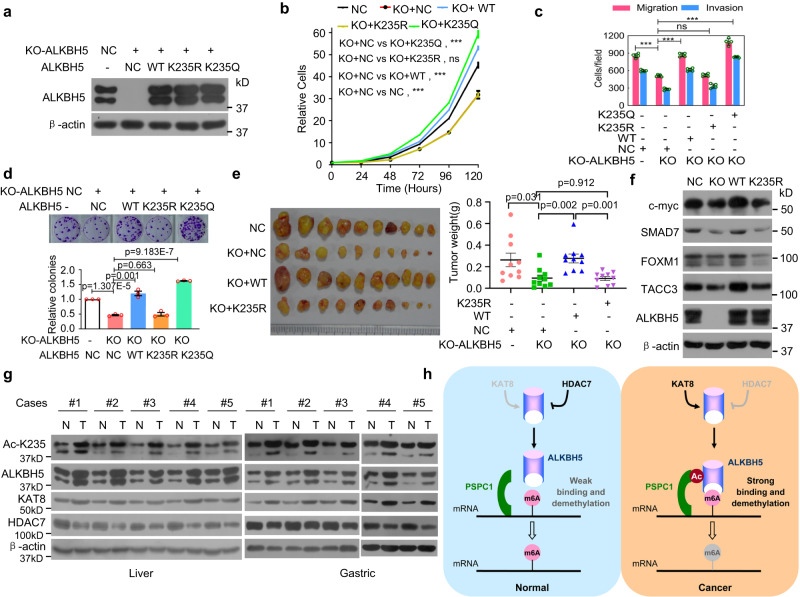
K235 acetylation of ALKBH5 is upregulated in cancers and is critical for the oncogenic roles of ALKBH5. **a**–**d** The ALKBH5 protein level (**a**); cell proliferation (**b**) (*n* = 3); and migration, invasion (**c**) (*n* = 5), and colony formation (**d**) (*n* = 3) were determined in ALKBH5 KO HeLa cells stably re-expressing wild-type ALKBH5 or its mutants K235R or K235Q. **e** The in vivo tumorigenesis of the indicated ALKBH5 KO HeLa cells stably re-expressing wild-type ALKBH5 or its mutant K235R was examined, and the weights of the xenograft tumors were analyzed (n = 10 mice per group). **f** The levels of the indicated proteins were determined in ALKBH5 KO HeLa cells stably reexpressing wild-type ALKBH5 or its mutant K235R. **g** K235 acetylation and ALKBH5, KAT8, and HDAC7 levels were determined in ten pairs of fresh liver and gastric cancer tissues and their corresponding nontumor tissues. **h** A regulatory model of ALKBH5 m^6^A demethylation activity is elucidated in which K235-acetylated ALKBH5 primarily functions as the catalytic core, and PSPC1 serves as an RNA-binding platform to recruit and facilitate the recognition of RNA m^6^A by ALKBH5 by interacting with K235-acetylated ALKBH5, thereby promoting RNA m^6^A erasure. Two-tailed unpaired Student’s *t* test in **c**–**e** and two-way ANOVA in (**b**). The data are represented as the mean ± SD. **p < 0.01, ***p < 0.001, ns indicates no significance. Source data are provided as a Source Data file.

Mitogenic signals play critical roles in cell proliferation and tumorigenesis. Previous studies have shown that ALKBH5 promotes tumorigenesis^14,16–18,27^. Therefore, the mitogenic signal that stimulated ALKBH5 K235 acetylation led us to speculate that ALKBH5 K235 acetylation affects tumorigenesis. KO of ALKBH5 inhibited cancer cell proliferation, colony formation, migration, and invasion in HeLa cells (Fig. 9a–d). Similar results were obtained in HCT-116 cells in which ALKBH5 expression was knocked down (Supplementary Fig. 10). The ALKBH5 KO-induced alterations in cell proliferation, colony formation, migration, and invasion were restored to control levels after re-expression with wild-type ALKBH5 and K235Q but not after re-expression with K235R ALKBH5 (Fig. 9a–d). Similar results were obtained in HCT-116 cells in which wild-type ALKBH5 and its mutants K235R and K235Q, which were resistant to anti-ALKBH5 siRNA, were re-expressed after cellular ALKBH5 expression was silenced by anti-ALKBH5 siRNA (Supplementary Fig. 10).

Furthermore, HeLa cells stably re-expressing wild-type ALKBH5 or its mutant K235R in the ALKBH5 KO HeLa cell line were constructed. The tumors that arose in mice injected with ALKBH5 KO cells were much smaller than those in mice that were injected with NC cells in which ALKBH5 was not knocked out. The in vivo xenograft tumor growth with ALKBH5 KO cells stably re-expressing wild-type ALKBH5 was restored to that with NC cells, whereas the in vivo xenograft tumor growth with ALKBH5 KO cells stably re-expressing the K235R mutant was not restored to that with NC cells and was similar to that with ALKBH5 KO cells (Fig. 9e).

The differential m^6^A peaks between ALKBH5 WT and K235R (*p* ≤ 0.01 and logFC ≥1) were collected into a total of 1012 genes, including FOXM1, c-myc, SMAD7, and TACC3, which have been reported to be the m^6^A targets mediated by ALKBH5 in tumorigenesis. GO analyses showed that the 1012 genes were involved in system development, cell morphogenesis, cell differentiation, metabolic regulation, cytoskeleton organization, cell adhesion, cell migration, and immune regulation, which are related to tumorigenesis. As previously reported, KO of ALKBH5 decreased the protein levels of FOXM1, c-myc, SMAD7, and TACC3 (Fig. 9f). The ALKBH5 KO-induced alterations of the FOXM1, c-myc, SMAD7, and TACC3 levels were restored to the control levels after re-expression with wild-type ALKBH5 but not after re-expression with the ALKBH5 K235R mutant (Fig. 9f).

Finally, we determined the levels of K235 acetylation and ALKBH5, KAT8, and HDAC7 in ten pairs of matched fresh primary liver and gastric tumor samples and adjacent nontumor liver and gastric tissues. The K235 acetylation level of ALKBH5 was substantially higher in the primary liver and gastric tumors than that in their corresponding adjacent nontumor liver and gastric tissues. The ALKBH5 and KAT8 levels were also higher in tumors than those in their corresponding nontumor tissues, whereas the HDAC7 level was lower in tumors than that in their corresponding nontumor tissues (Fig. 9g). Collectively, K235 acetylation is critical for the oncogenic roles of ALKBH5.

## Discussion

ALKBH5 is one of two RNA m^6^A demethylases^3,14^. Our findings reveal that the RNA m^6^A demethylation activity of ALKBH5 is orchestrated by its K235 acetylation and its regulatory subunit PSPC1 (Fig. 9h). K235 acetylation of ALKBH5 promotes its m^6^A demethylation activity by increasing the binding and recognition of ALKBH5 to RNA m^6^A, thereby strengthening the removal of m^6^A from RNAs. K235 acetylation strengthens the m^6^A demethylation activity of ALKBH5 by increasing the binding of ALKBH5 itself to RNA m^6^A. In addition, K235-acetylated ALKBH5 preferentially binds to the RNA-binding protein PSPC1, which further facilitates the recruitment and recognition of ALKBH5 to RNA m^6^A, thereby facilitating the removal of RNA m^6^A modification by ALKBH5.

Limited acetylation was detected in ALKBH5 mutants K235R and K235Q using the anti-pan acetylated lysine antibody, suggesting that other acetylation sites existed on ALKBH5 in addition to the K235 acetylation site. The K132 acetylation site has been reported in an acetylome profiling database^28^. The influences of K132 acetylation on ALKBH5 function or activity will be valuable to investigate in the future. Whether the activity of other methyltransferases or demethylases in the m^6^A pathway is also regulated by acetylation modifications will also be valuable to explore in the future.

In this study, we found that the RNA-binding protein PSPC1 is a regulatory subunit of ALKBH5. PSPC1 preferentially binds to K235-acetylated ALKBH5 and increases the demethylase activity of ALKBH5 by facilitating the recognition and binding of ALKBH5 to RNA m^6^A. PSPC1 itself did not alter the RNA m^6^A level based on an in vitro demethylation assay or alter the m^6^A level in ALKBH5 KO cells, indicating that the regulatory subunit PSPC1 is not a catalytic subunit of the ALKBH5 m^6^A demethylase complex. Previous studies have shown that PSPC1 is an RNA-binding protein and binds to RNA through its two RRM domains (RRM1 and RRM2)^29^. When the binding of PSPC1 to RNAs is eliminated, PSPC1 does not strengthen the binding of ALKBH5 to RNA m^6^A and thus does not strengthen the m^6^A demethylation activity of ALKBH5. The binding of PSPC1 to both RNAs and K235-acetylated ALKBH5 facilitates the recruitment and binding of ALKBH5 to RNA m^6^A, thereby facilitating m^6^A removal from RNAs by ALKBH5. Therefore, a regulatory model of the RNA m^6^A demethylase ALKBH5 eraser complexes is elucidated here in which ALKBH5 functions as the catalytic core, PSPC1 serves as an RNA-binding platform to recruit and facilitates the recognition of RNA m^6^A by ALKBH5 through the interaction with K235-acetylated ALKBH5, and K235 acetylation provides a link between ALKBH5 and its regulatory subunit PSPC1.

PSPC1 is found in paraspeckles, which serve as dynamic molecular scaffolds for protein-protein and protein-nucleic acid interactions to participate in the regulation of transcriptional regulation, RNA processing, RNA transport, A-to-I editing, and DNA repair^20–22^. PSPC1 forms dimers and polymers with other RNA-binding proteins such as NONO and SFPQ^20^. As expected, some PSPC1 binds to NONO and SFPQ to form a functional complex, whereas some PSCP1 binds to K235-acetylated ALKBH5 to form another functional complex, which plays a critical role in RNA m^6^A demethylation.

Surprisingly, the m^6^A distribution showed a higher start codon density for the K235R mutant than for wild-type ALKBH5 (Fig. 4f). The cause of the distribution difference may be that in addition to binding to PSPC1, ALKBH5 regulates m^6^A demethylation in different regions of RNA, possibly by binding to different partners (subunits), which preferentially bind to different regions of RNA such as the 3′UTR, CDS or 5′UTR, indicating that other regulatory subunits or partners of ALKBH5 or K235-acetylated ALKBH5 exist. Wild-type ALKBH5 and ALKBH5 K235R mutants may bind to different partners to remove RNA m^6^A in different RNA regions. This phenomenon will be valuable for further investigation in the future.

ALKBH5 is upregulated in cancers and plays oncogenic roles as an m^6^A demethylase in glioblastoma, acute myeloid leukemia, gastric cancer, breast cancer, and ovarian cancer^14,16,17^. We found that ALKBH5 expression and the levels of K235 acetylation of ALKBH5 and its acetyltransferase KAT8 are increased and its deacetylase HADC7 level is downregulated in liver and gastric cancers. KO and KD of ALKBH5 inhibited cancer cell proliferation, colony formation, migration, and invasion in vitro and tumorigenesis in vivo. K235-acetylated ALKBH5, but not non-K235-acetylated ALKBH5, exerts oncogenic functions, consistent with the results in which mitogenic signals stimulate K235 acetylation of ALKBH5 and cell growth. Therefore, we revealed that K235 acetylation is critical for the oncogenic roles of ALKBH5.

In summary, ALKBH5 is acetylated at K235 by the acetyltransferase KAT8 and deacetylated by the deacetylase HDAC7. K235 acetylation is critical for the m^6^A demethylation activity of ALKBH5 by increasing the recognition and binding of ALKBH5 to RNA m^6^A. The RNA-binding protein PSPC1 is a regulatory subunit of ALKBH5 and preferentially interacts with K235-acetylated ALKBH5, recruits ALKBH5 to RNA m^6^A and further facilitates the recognition of RNA m^6^A by ALKBH5, thereby promoting m^6^A erasure. Mitogenic signals stimulate K235 acetylation of ALKBH5. K235 acetylation is upregulated in cancers and critical for the m^6^A demethylation activity and oncogenic functions of ALKBH5.

## Methods

The collection of the cancer samples was approved by the Internal Review and Ethics Boards at the Third Affiliated Hospital of Guangzhou Medicine University. Informed consent was obtained from each patient.

### Cell culture and tissue samples

HCT-116, HeLa, and HEK293T cell lines were obtained from the American Type Culture Collection (ATCC, USA) and cultured under standard conditions. The cell lines were authenticated by ATCC via STR profiling. All cell lines tested negative for mycoplasma contamination. Fresh-frozen primary liver and gastric cancer tissues and matched adjacent nontumor samples of liver and gastric tissues were collected from cancer patients at the Affiliated Hospital of Guangzhou Medical University. These cases were selected based on a clear pathological diagnosis, and patients with liver or gastric cancer were not preoperatively treated with anticancer agents. The collection of these samples was approved by the Internal Review and Ethics Boards at the Third Affiliated Hospital of Guangzhou Medicine University. Informed consent was obtained from each patient.

### Plasmid constructs

The ALKBH5-FLAG, ALKBH5-HA, PSPC1-HA, and PSPC1-FLAG plasmids were generated as previously described^30^. The ALKBH5-FLAG mutants K235R and K235Q and the PSPC1mut mutant (F118A/F120A/K197A/F199A) were generated by a MutExpress II Fast Mutagenesis Kit V2 (Vazyme, China). The plasmids of all six acetyltransferases and all eighteen deacetylases were kindly provided by Prof. Tiebang Kang at Sun Yat-sen University Cancer Center, China.

### Construction of synonymous mutants

Synonymous mutant vectors of wild-type ALKBH5-FLAG and its mutants K235R and K235Q, which are resistant to anti-ALKBH5 siRNA, were constructed. The anti-ALKBH5 siRNA-targeted sequence 5′-GCTTCAGCTCTGAGAACTA-3′ in the ALKBH5-FLAG, K235R, and K235Q vectors was synonymously mutated to 5′-GTTTTAGTTCGGAAAATTA-3′. Synonymous mutant vectors of PSPC1-HA and KAT8-FLAG, which are resistant to anti-PSPC1 and KAT8 siRNA, respectively, were constructed. The anti-PSPC1 siRNA#1-targeted sequence 5′-GCACGAAAGGCTCTGGAAAGA-3′ in the PSPC1-HA vector was synonymously mutated to 5′-GCTCGTAAAGCACTCGAGCGA-3′. The anti-PSPC1 siRNA#2-targeted sequence 5′-ATGCTAATGAGGCAAGATCTA-3′ in the PSPC1-HA#2 vector was synonymously mutated to 5′-ATGTTGATGAGACAGGACTTG−3′. The anti-KAT8 siRNA-targeted sequence 5′-GGGAAAGAGATCTACCGCAAG-3′ in the KAT8-FLAG vector was synonymously mutated to 5′-GGAAAGGAAATATATCGTAAA−3′. Synonymous mutants were generated for wild-type sALKBH5-FLAG and its K235R and K235Q mutants as well as for sPSPC1-HA#1, sPSPC1-HA#2, and sKAT8-FLAG.

### Anti-acetyl-ALKBH5 (K235) antibody production

The epitope acetyl-peptide and nonacetyl-peptide were synthesized, and the anti-acetyl-ALKBH5 antibody was produced by GL Biochem (Shanghai), Ltd. Briefly, the KLH-coupled peptide GCKFQFK(Ac)RIRVSEP was synthesized, and polyclonal antibodies against acetyl-ALKBH5 (K235) were obtained from inoculated rabbits. Anti-non-K235-acetylated ALKBH5 antibody in the produced antibody was removed using affinity chromatography on columns containing the nonacetyl-peptide epitope GCKFQFKRIRVSEP. The anti-K235-acetylated ALKBH5 antibody was then further purified using affinity chromatography on columns containing the corresponding epitope acetyl-peptides.

### Western blotting

Total proteins from cells or tissues were prepared, and the protein concentrations were determined. Total proteins were separated using 10-12.5% SDS‒PAGE and then electroblotted onto a PVDF membrane. The indicated proteins were detected using the following antibodies: anti-Ac-K235 (developed in our lab, 1:500), acetylated lysine (Pan-Ac) (9814, CST, RRID:AB_10544700, 1:1000), ALKBH5 (703570, Thermo Fisher Scientific, RRID: AB_2762417, 1:1000), FLAG (M185-3 L, MBL, RRID: AB_11123930, 1:2000), HA (561, MBL, RRID: AB_591839, 1:2000), V5 (66007-1-Ig, Proteintech, RRID: AB_2734694, 1:1000), KAT8 (ab200660, Abcam, 1:1000), HDAC7 (33418, CST, RRID: AB_2716756, 1:1000), PSPC1 (16714-1-AP, Proteintech, RRID: AB_2878302, 1:1000), m^6^A (for dot blotting, ABE572, Merck Millipore, 1:1000), m^6^A (for m^6^A-seq, 202003, Synaptic systems, PRID: AB_2279214, 1:500), GST (2625 S, CST, RRID: AB_490796, 1:1000), NONO (11058-1-AP, Proteintech, RRID: AB_2152167, 1:1000), SFPQ (15585-1-AP, Proteintech, RRID: AB_10697653, 1:1000), FOXM1 (13147-1-AP, Proteintech, RRID:AB_2106213, 1:1000), SMAD7 (25840-1-AP, Proteintech, RRID:AB_2848137, 1:1000), TACC3 (25697-1-AP, Proteintech, RRID:AB_2880199, 1:1000), c-myc (13987 S, CST, RRID:AB_2631168, 1:1000) and β-actin (60008-1-Ig, Proteintech, RRID: AB_2289225, 1:5000).

The anti-Ac-K235 antibody developed in our laboratory was validated by our group. The related data are provided in Supplementary Fig. 2. All of the commercially available antibodies used in this study were validated for use in human specimens by the manufacturers and for the respective methods used in this study.

### Immunoprecipitation (IP) and coimmunoprecipitation (co-IP) assays

IP and co-IP assays were performed as previously described^31^. IP or co-IP experiments were performed using anti-FLAG, anti-HA or anti-V5 antibodies, and the immune complexes were captured on Protein A/G agarose beads (Santa Cruz). The immunoprecipitated ALKBH5-FLAG was separated by SDS‒PAGE, and the gel was stained with Coomassie Blue for mass spectrometry identification. The coimmunoprecipitated complexes were separated, and the gels were stained with silver for a mass spectrometry assay or used for a western blotting assay with the indicated antibodies. For the mass spectrometry assay, two independent experiments were performed with a differential gel band and its corresponding negative gel band excised and in-gel-digested with trypsin.

### Mass spectrometry analyses

Protein acetylation and protein identification experiments were performed by mass spectrometry as previously described with minor modifications^32^. The mass spectrometry assay was performed by Bioinnovation Bio., Shenzhen.

For the identification of ALKBH5 acetylation sites, the extracted peptide mixtures were dissolved in a buffer containing 0.1% formic acid and 2% acetonitrile (AcN) and analyzed using nano-LC‒MS/MS (AB SCIEX Triple TOF 6600, USA). The WIFF RAW files were converted into peak list files using PEAKS Studio 8.5 (Bioinformatics Solutions Inc. Waterloo, Canada). ALKBH5 acetylation site identification was performed using the Mascot (v2.3.02) program against the UniProt human protein database (released Dec 2014) with the default parameters. Carbamidomethyl (C) was set as a fixed modification, whereas acetyl (protein N-terminus), dehydrated (NQ), and oxidation (M) were considered variable modifications. The FDR was 5%.

To identify the ALKBH5 interactors, the extracted peptide mixtures were dissolved in a buffer containing 0.1% formic acid and 2% acetonitrile (AcN) and analyzed using nano-LC‒MS/MS (Q Exactive, Thermo Scientific, USA). The RAW files were converted into peak list files using PEAKS Studio 8.5. Protein identification was performed using the Mascot (v2.3.02) program against the UniProt human protein database (released Dec 2014) with the default parameters. The FDR was 1%, and the unique peptides were ≥2.

The mass spectrometry proteomics data for the identification of ALKBH5 acetylation and K235-acetylated ALKBH5-interaction partners have been deposited in the ProteomeXchange Consortium via the iProX partner repository^33^ with the dataset identifiers PXD020070 and PXD020071, respectively.

### Generation of ALKBH5 KO cell lines using CRISPR‒Cas9

KO of ALKBH5 was performed using CRISPR‒Cas9 as previously described^13^. The ALKBH5 Cas9/sgRNA vector pGE-4 (pU6-gRNA1 Cas9-puroU6-gRNA2) was constructed by GenePharma (Shanghai, China). The two sgRNA targeting sites in ALKBH5 were 5′-GACGTCCCGGGACAACTATA-3′ (site 1) and 5′-TACCCTGTGTCCGGGGCCAA-3′ (site 2). After HeLa cells were transfected with the vector using Lipofectamine 2000 (Invitrogen) for 24 h, these cells were selected with puromycin for 36 h. The cells were then diluted and seeded onto 96-well plates for single-cell culture to obtain single cell-derived colonies. ALKBH5 KO was confirmed by PCR and sequencing. ALKBH5 KO HeLa cell colonies were selected, cultured and saved for subsequent use.

### RNA interference

The anti-ALKBH5, anti-KAT8, anti-HDAC7, or anti-PSPC1 siRNAs or negative control (NC) siRNA (GenePharma) were transfected into the cells with RNAiMAX (Invitrogen) for 48 h (unless otherwise stated). Together with siRNAs, the vectors were cotransfected using Lipofectamine 2000 (Invitrogen) for 48 h (unless otherwise stated). The siRNA sequences are provided in Table S1.

### In vitro acetylation assay

Wild-type ALKBH5-FLAG and its mutant K235R-FLAG or KAT8-FLAG plasmids were transfected into HEK293T cells, and ALKBH5-FLAG, ALKBH5-FLAG K235R, and KAT8-FLAG proteins were purified according to the kit instructions (FLAGIPT1, Sigma). The in vitro deacetylation assay was performed as previously described with minor modifications^34^. Briefly, immunopurified wild-type ALKBH5 or K235R was incubated with immunopurified KAT8 in HAT buffer (Millipore) in a 30 °C shaking incubator for 1 h. The K235 acetylation of ALKBH5 was determined by western blotting using anti-Ac-K235 and pan-acetylated lysine antibodies.

### In vitro deacetylation assay

The ALKBH5-FLAG plasmid was transfected into HEK293T cells together with the KAT8 vector, which was used to induce K235 acetylation of ALKBH5. The HDAC7-FLAG plasmid was transfected into HEK293T cells. ALKBH5-FLAG and HDAC7-FLAG proteins were purified according to the kit instructions (FLAGIPT1, Sigma). The in vitro deacetylation assay was performed as previously described with minor modifications^34^. Immunopurified ALKBH5-FLAG containing Ac-K235-ALKBH5 was incubated with immunopurified HDAC7 in HEPES buffer (40 mM HEPES, 1 mM MgCl_2_, 1 mM DTT, and 5 mM NAD^+^) at 37 °C for 1 h. The K235 acetylation of ALKBH5 was determined by western blotting using anti-Ac-K235 and pan-acetylated lysine antibodies.

### RNA m^6^A dot blotting

RNA m^6^A dot blotting was performed as previously described^13^. Briefly, mRNA was purified from total RNA using a Dynabeads mRNA Purification Kit (Thermo Fisher Scientific). mRNA was denatured in a denaturing buffer (2.2 M formaldehyde, 50% deionized formamide, and 0.5х MOPS buffer) at 55 °C for 15 min. mRNA was heated at 95 °C for 10 min and cooled on ice for 5 min. These mRNAs were then spotted onto Amersham Hybond-N+ membranes (GE Healthcare, cat# RPN303B) and crosslinked by UV light. mRNA m^6^A was detected with an anti-m^6^A antibody. The loaded mRNAs were stained with methylene blue (MB).

### Quantitative measurement of the RNA m^6^A level by LC‒MS/MS

Quantitative analysis of the RNA m^6^A level was performed as previously described with minor modifications^3,13^. Briefly, the purified mRNA and synthetic RNA-oligonucleotide substrates were digested into single nucleosides in a buffer (0.01 U phosphodiesterase I, 1 U nuclease P1, 2 mM zinc chloride, and 30 mM sodium acetate at pH 6.8) for 3 h at 37 °C and dephosphorylated with 10 U bacterial alkaline phosphatase for 1 h at 37 °C. Enzymes were removed by filtration. Individual nucleosides were separated by liquid chromatography coupled with tandem mass spectrometry (LC‒MS/MS) on a Hypersil GOLD aQ reversed-phase column (Thermo Scientific) using an Agilent 6490 Triple Quadrupole mass spectrometer. Nucleosides were quantified using nucleoside-to-base ion mass transitions of 252.1 to 136.1 for A and 282.1-150.1 for RNA m^6^A. The m^6^A and A concentrations were measured by comparison with the standard curves obtained from their nucleoside standards. The ratio of m^6^A to A was determined based on the calculated concentrations.

### m^6^A-seq and data analysis

m^6^A-seq was performed by LC-BIO Biotech, Inc. (Hangzhou, China) according to the reported protocol with minor modifications^7,14^. Briefly, total RNA was extracted using TRIzol reagent (Invitrogen, CA, USA) following the manufacturer’s procedure. mRNA was purified using a poly-T oligo attached to magnetic beads (Invitrogen). mRNA was then fragmented into ~100 nt oligonucleotides using divalent cations under an elevated temperature. Fragmented mRNA was incubated with an m^6^A-specific antibody in IP buffer (50 mM Tris-HCl, 750 mM NaCl and 0.5% Igepal CA-630) supplemented with 0.5 μg/μl BSA for 2 h at 4 °C. Eluted m^6^A-containing fragments (IP) and untreated input control fragments (Input) were used for library preparation. Sequencing was carried out on an Illumina NovaSeq 6000 platform according to the manufacturer’s instructions.

All reads were mapped to the human genome (Version GRCh38.p12) by bowtie using default settings^35^. Mapped reads of m^6^A fragments and input libraries were provided through the R package exomePeak^36^, which identifies m^6^A peaks with a bed or bam format that can be adapted for visualization on the UCSC genome browser (https://genome.ucsc.edu/cgi-bin/hgGateway) using IGV software (http://www.igv.org/). The HOMER program was used to identify de novo and known motifs^37^.

The m^6^A-seq data of K235 acetylation-mediated m^6^A profiles have been deposited into the Gene Expression Omnibus (GEO) under accession number GSE142203. The m^6^A-seq data of ALKBH5- and PSPC1-mediated m^6^A profiles have been deposited in the Genome Sequence Archive (GSA) of the BIG Data Center, Beijing Institute of Genomics (BIG, http://gsa.big.ac.cn) under accession number HRA000565.

### Recombinant ALKBH5 and PSPC1 protein production

GST-ALKBH5 and PSPC1-His were cloned into the pGEX and pET 26b vectors, respectively. These vectors were transferred into BL21 (DE3) E. coli, and GST-ALKBH5, K235R mutant, or PSPC1-His expression was induced by 0.5 mM IPTG for 4-5 h. The E. coli were then collected, lysed using extraction buffer (20 mM HEPES, pH 7.6; 0.5 M NaCl; 0.5 μM EDTA; 10% glycerol; and 0.5% NP-40), and ultrasonicated. The supernatant fractions were then collected. Recombinant GST-ALKBH5 or K235R mutant protein beads were obtained from the supernatant fractions using Glutathione Sepharose (Millipore, G0924), and recombinant GST-ALKBH5 or its K235R mutant were eluted by L-glutathione reduction (Sigma, G4251). Recombinant PSPC1-His was purified from the supernatant fractions using a His-tag Protein Purification Kit (denaturant-resistant) (Beyotime, P2229S).

### GST pull-down assay

The recombinant GST-ALKBH5 protein beads were incubated with recombinant PSPC1-His at 4 °C for 1 h. The beads were washed three times using washing buffer (20 mM HEPES, pH 7.6; 0.1 M KCl; 0.1 μM EDTA; 10% glycerol; and 0.02% NP-40). GST-ALKBH5 complexes were eluted from the beads by l-glutathione reduction. PSPC1-His and GST-ALKBH5 were detected using western blotting assays.

### In vitro ALKBH5 and/or PSPC1 demethylation activity assay

The in vitro ALKBH5 and/or PSPC1 demethylation activity assay was performed as previously reported with minor modifications^13,14^. Briefly, the reactions were performed in a 5 μl demethylation reaction buffer containing 50 pmol m^6^A RNA oligos, 500 ng immunopurified wild-type ALKBH5-FLAG, ALKBH5-FLAG K235 and/or immunopurified PSPC1-FLAG protein (or recombinant ALKBH5 and/or recombinant PSPC1), 50 mM HEPES buffer (pH 7.0), 100 mM KCl, 2 mM MgCl_2_, 1.6 U/μl RNasin (Takara), 2 mM L-ascorbic acid, 300 μM α-ketoglutarate, and 150 μM (NH_4_)_2_Fe(SO_4_)_2_·6H_2_O. The reaction was incubated at room temperature for 1 h and then quenched by adding 5 μl denaturing buffer (2.2 M formaldehyde, 50% deionized formamide, and 0.5х MOPS buffer) followed by heating at 95 °C for 10 min. Next, 2 μl of the reaction product was used for m^6^A dot blotting. The m^6^A-oligo sequence was 5′-AUUGUGGm^6^ACUGCAGC-3′.

### RNA affinity purification

Biotin-labeled RNA oligos containing A or m^6^A were synthesized by GeneScript (China). RNA affinity purification was performed as previously described^31,38^. Briefly, 1 nmol of biotin-labeled A- or m^6^A-RNA oligos was bound to 100 μl of Streptavidin Agarose beads (Sigma) overnight at 4 °C with rotation. RNA-immobilized beads were mixed with whole cell extracts or recombinant ALKBH5 and PSPC1 and incubated at 30 °C for 30 min. These beads were eluted by adding 30 μl of protein loading buffer and boiling for 5 min. ALKBH5 and/or PSPC1 in the eluted mixtures were then detected using western blotting. The A and m^6^A oligo sequences were 5′-CGUCUCGGACUCGGACUGCU-3′ and 5′-CGUCUCGGm^6^ACUCGGm^6^ACUGCU-3′, respectively.

### Cell proliferation, colony formation, and migration and invasion assays

Cell proliferation, colony formation, and migration and invasion assays were performed as previously described^31,39^. Briefly, ALKBH5 KO HeLa cells were transfected with the indicated ALKBH5 plasmids for 12 h, or HCT-116 cells were cotransfected with anti-ALKBH5 siRNA together with the indicated ALKBH5 plasmids for 12 h. For the cell proliferation assay, 1 × 10^4^ cells were then plated in 96-well culture plates and cultured. The cell number was counted at 24, 48, 72, 96, and 120 h (*n* = 3). For the cell colony formation assay, 250 cells were then plated in 6-well culture plates and cultured in a medium supplemented with 10% FBS. These cells were then fixed with methanol and stained with crystal violet solution. The colony numbers were counted under a microscope (*n* = 3). For the migration and invasion assays, 1 × 10^5^ cells in 100 μl cell suspensions with 0.05% FBS were added to the upper Transwell chambers for the migration assay (8.0 μM pore size, BD) or the upper Transwell chambers coated with Matrigel for the invasion assay, and medium with 10% FBS was added to the bottom chamber. Migrated and invasive cells were stained with 5% crystal violet. Images were obtained from each membrane and migrated and invasive cells were counted under a microscope.

### Construction of cell lines with stable expression of ALKBH5 or K235R

ALKBH5 KO HeLa cells were infected with lentiviruses expressing wild-type ALKBH5-FLAG or its mutant K235R and selected using puromycin. The ectopic expression of ALKBH5 was validated by western blotting. ALKBH5 KO HeLa cell lines stably expressing wild-type ALKBH5-FLAG (KO + WT) and ALKBH5-FLAG K235R (KO + K235R) were established.

### In vivo tumor growth in a mouse model

The in vivo tumor growth in the mouse model was performed as previously described^31,38^. Female BALB/c nude mice (3–4 weeks old) were purchased from Guangdong Medical Animal Experiment Center (Guangzhou, China). Briefly, the indicated HeLa cells (1 × 10^6^) were injected subcutaneously into the right or left armpit of each mouse to establish a tumor xenograft model (*n* = 10). After two weeks, these mice were euthanized by injecting sodium pentobarbital, and the tumor xenografts were excised and weighed.

The mice used in these experiments were bred and maintained under defined conditions (at room temperature (20-26 °C) and 40-70% humidity with a 12 h light/dark cycle and access to food and water ad libitum) at the Guangdong Medical Animal Experiment Center (SPF-grade facility). The animal experiments were approved by the Laboratory Animal Ethics Committee of the Third Affiliated Hospital of Guangzhou Medicine University and Guangdong Medical Animal Experiment Center and conformed to the legal mandates and national guidelines for the care and maintenance of laboratory animals. The maximal tumor size permitted by this ethics committee should not exceed 20 mm in any one dimension. In this study, the maximal tumor size was not exceeded 20 mm in any one dimension.

### Statistics and reproducibility

Statistical analyses were performed using Prism 8 software and the SPSS program. Two-tailed, unpaired Student’s *t*-tests were used to compare data between two groups. A two-way ANOVA was used to analyze the significance of the growth curves. The treatment groups were compared with the control unless stated otherwise. At least three independent experiments were performed in Figs. 4b, 6b, 9b–e and supplementary Figs. 4 and 10b–d (The numbers (n) of the independent experiments were indicated in Figure legends). At least two biologically independent experiments were performed with similar results and representative results are shown in Figs. 1–3, 4a, c–j, 5, 6c–h, 7, 8, 9a, f and g, supplementary Figs. 1–3, 5–9 and 10a. The data are presented as the mean ± SD unless otherwise indicated. Differences of *p < 0.05, **p < 0.01 or ***p < 0.001 were considered statistically significant; ns, indicates no statistical significance.

### Reporting summary

Further information on research design is available in the Nature Portfolio Reporting Summary linked to this article.

## Supplementary information

Supplementary Information

Reporting Summary

## Data Availability

The m^6^A-seq data of K235 acetylation-mediated m^6^A profiles have been deposited into the Gene Expression Omnibus (GEO) under accession number GSE142203 (https://www.ncbi.nlm.nih.gov/geo/query/acc.cgi?acc=GSE142203). The m^6^A-seq data of ALKBH5- and PSPC1-mediated m^6^A profiles have been deposited in the Genome Sequence Archive (GSA) of the BIG Data Center, Beijing Institute of Genomics (BIG, http://gsa.big.ac.cn) under accession number HRA000565 (https://bigd.big.ac.cn/gsa-human/browse/HRA000565). The mass spectrometry proteomics data on the identification of ALKBH5 acetylation and K235-acetylated ALKBH5-interaction partners have been deposited in the ProteomeXchange Consortium via the iProX partner repository^33^ with the dataset identifiers PXD020070 and PXD020071, respectively. The human genome database (Version GRCh38.p12) was freely downloaded from Ensemble (www.ensembl.org). All data are available in the main article and supplementary information. Source data are provided as a Source Data file. Source data are provided with this paper.

## Source data

Source Data
