# Supplementary material for: K235 acetylation couples with PSPC1 to regulate the m6A demethylation activity of ALKBH5 and tumorigenesis
Source: Nat Commun. 2023 Jun 27;14:3815. doi: 10.1038/s41467-023-39414-4 (PMC10300122; doi:10.1038/s41467-023-39414-4)
Supplement: Supplementary file 1 — Supplementary Information [file 41467_2023_39414_MOESM1_ESM.pdf]

**Supplementary Information**

# K235 acetylation couples with PSPC1 to regulate the m$^6$A demethylation activity of ALKBH5 and tumorigenesis

Xiao-Lan Zhang, Xin-Hui Chen, Binwu Xu, Min Chen, Song Zhu, Nan Meng, Ji-Zhong Wang, Huifang Zhu, De Chen, Jin-Bao Liu, Guang-Rong Yan

This PDF file includes:

Supplementary Figure 1 to 10

Supplementary Figure Legends

Supplementary Table 1

**Supplementary Figures**

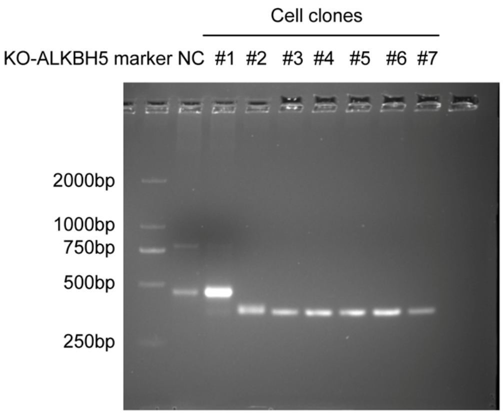


**Supplementary Figure 1.** *ALKBH5* KO cell lines were constructed. *ALKBH5* in HeLa cells was knocked out by CRISPR-Cas9, *ALKBH5* KO cell colonies were selected and the corresponding cell lines were established. Source data are provided as a Source Data file.

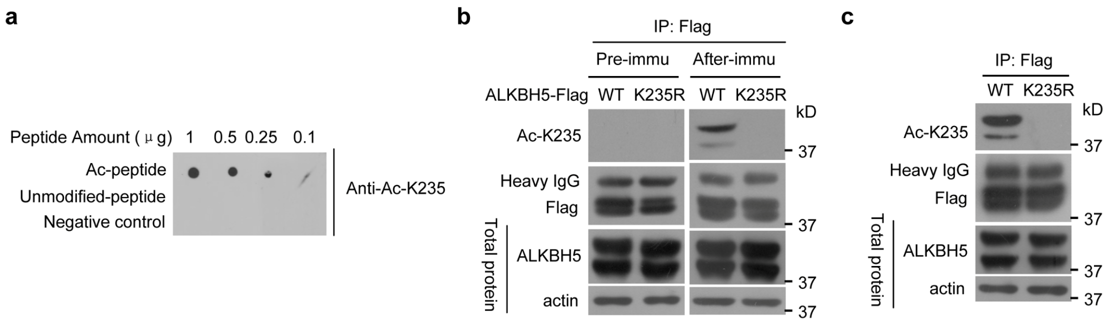


**Supplementary Figure 2.** Our developed antibody, Ac-K235, against anti-K235 acetylated ALKBH5 specifically detected K235-acetylated ALKBH5. (a) The Ac-K235 antibody specifically detected the acetylated K235 peptide. A nitrocellulose membrane was spotted with different amounts of the acetyl-K235 peptide (GCKFQFK(Ac)RIRVSEP), unmodified peptide (GCKFQFKRIRVSEP) or negative control peptide (KKQLEAEIIDF) and probed with the anti-Ac-K235 antibody. (b) HeLa cells were transfected with wild-type *ALKBH5-FLAG* or its mutant *K235R*, ALKBH5-FLAG was IPed using an anti-FLAG antibody, and K235 acetylation in IPed ALKBH5-FLAG was detected using preimmunity serum and after-immunity serum. (c) HeLa cells were transfected with wild-type *ALKBH5-FLAG* or its mutant *K235R*, ALKBH5-FLAG was IPed using an anti-FLAG antibody, and K235 acetylation in IPed ALKBH5-FLAG was detected using the Ac-K235 antibody. Source data are provided as a Source Data file.

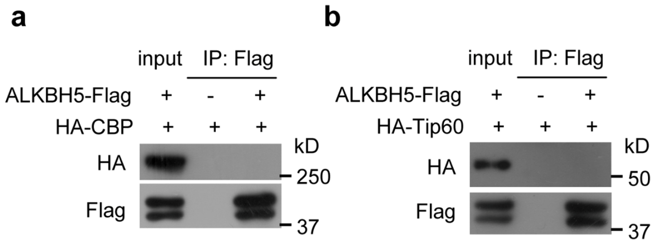


**Supplementary Figure 3.** ALKBH5 did not interact with other acetyltransferases such as CBP and Tip60. *ALKBH5-FLAG* plasmid together with *HA-CBP* (a) or *HA-Tip60* vector were cotransfected into HeLa cells, and ALKBH5-FLAG complexes were co-IPed using anti-FLAG antibody, and the HA-CBP (A) or HA-Tip60 (b) were detected. Source data are provided as a Source Data file.

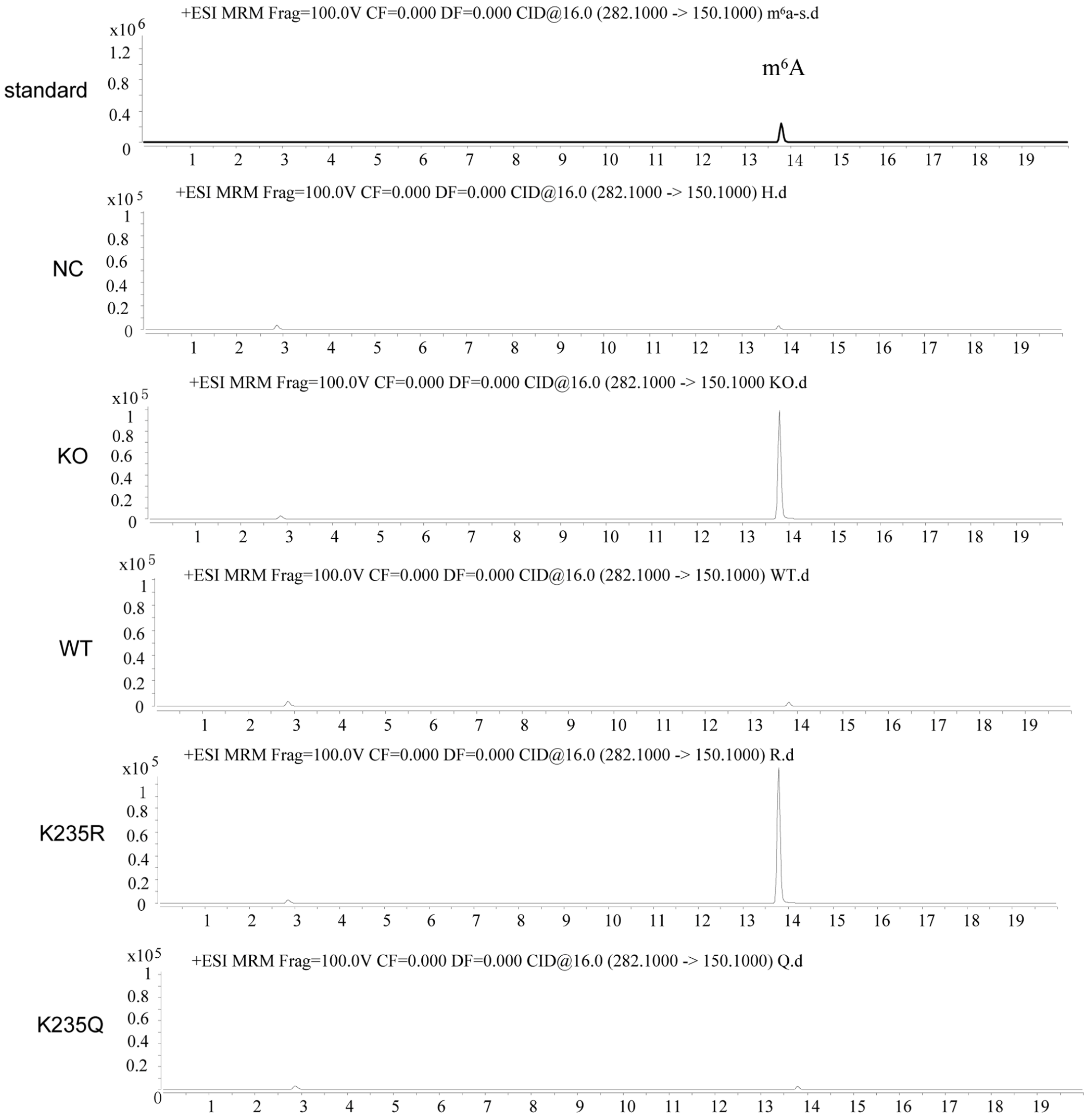


**Supplementary Figure 4.** RNA $m^6A$ levels mediated by wild-type ALKBH5 and its mutants K235R and K235Q were determined using LC-MS/MS assay. The wild-type *ALKBH5* or its mutants *K235R* and *K235Q* plasmid was transfected into *ALKBH5* KO cells, the purified mRNAs were used to detect RNA $m^6A$ using LC-MS/MS.

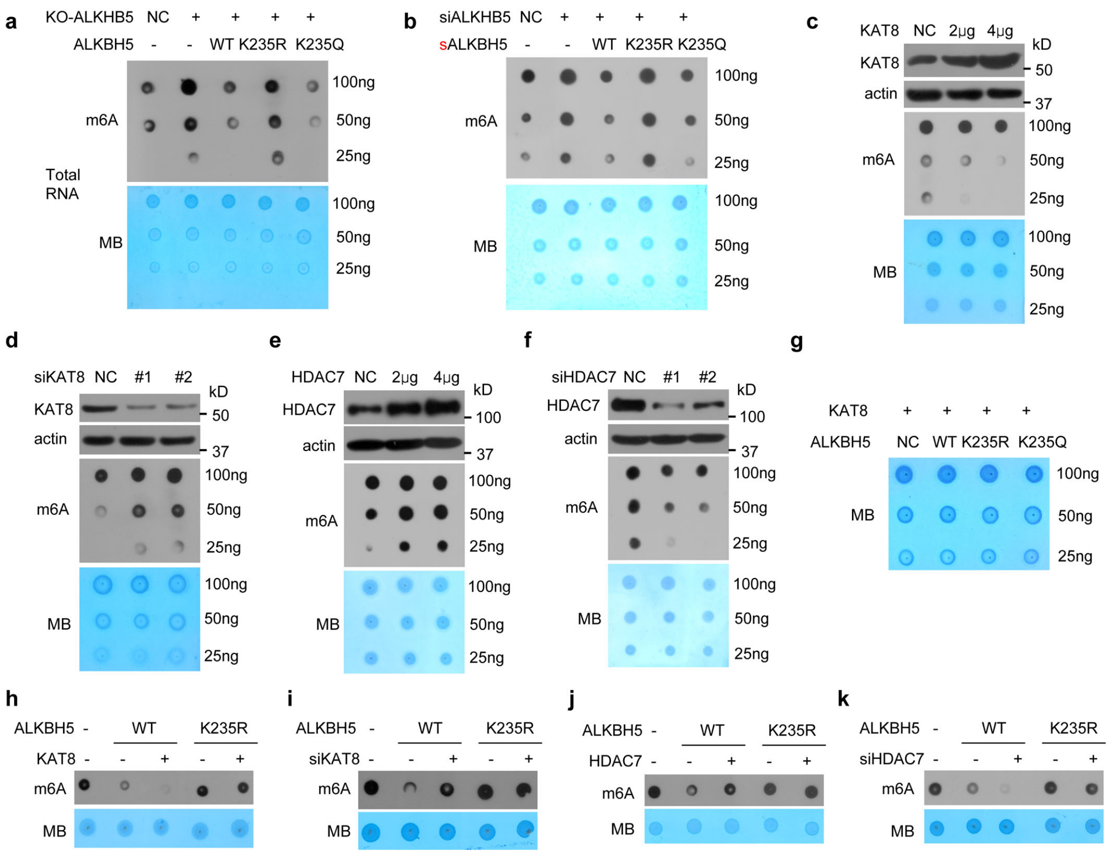


**Supplementary Figure 5.** K235 acetylation of ALKBH5 promotes the RNA m$^6$A demethylation activity of ALKBH5. (a) The wild-type *ALKBH5* and its mutant *K235R* and *K235Q* plasmids were transfected into *ALKBH5* KO HeLa cells, and the cellular total RNA m$^6$A level was determined by dot blotting. (b) The synonymously mutated wild-type *sALKBH5* and its mutant *K235R* and *K235Q* plasmids with anti-*ALKBH5* siRNA were cotransfected into HCT-116 cells, and the cellular mRNA m$^6$A levels were determined. (c) *KAT8* overexpression reduced cellular mRNA m$^6$A levels in a dose-dependent manner. (d) KD of *KAT8* increased cellular mRNA m$^6$A levels. (e) *HDAC7* overexpression increased cellular mRNA m$^6$A levels. (f) KD of *HDAC7* decreased cellular mRNA m$^6$A levels. (g) MB staining of the loaded RNA in Figure 4h as a loading control. (h-k) KAT8 plasmid (h), anti-KAT8 siRNA (i), HDAC7 plasmid (j) or anti-HDAC7 siRNA (k) was transfected into *ALKBH5* KO HeLa cells stably reexpressing the wild-type ALKBH5-FLAG or K235R mutant, wild type ALKBH5 or its K235R mutant was then purified. The purified wild-type ALKBH5 or its K235R mutant was incubated with m$^6$A RNA oligos, and the m$^6$A level was determined. Source data are provided as a Source Data file.

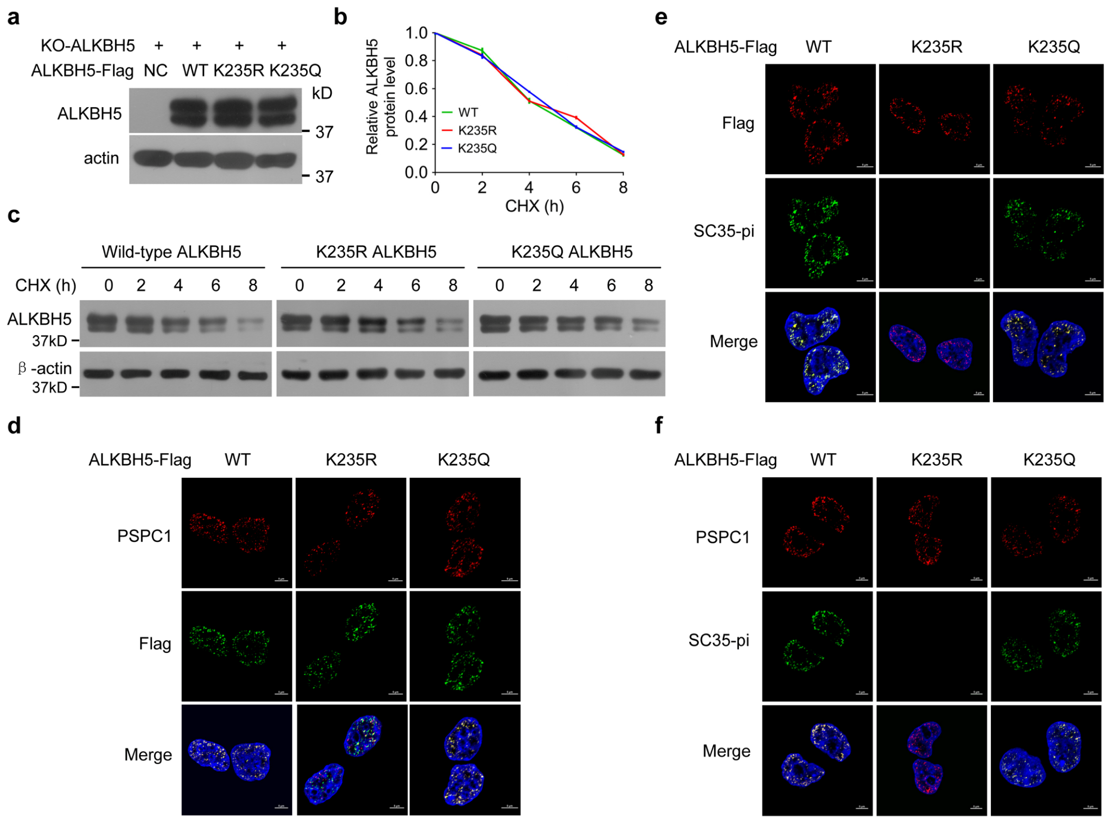


**Supplementary Figure 6.** ALKBH5 acetylation at K235 does not alter the level, stability and sublocalization of ALKBH5, K235 deacetylation of ALKBH5 impairs the interaction of ALKBH5 with PSPC1 and the disappearance of the nuclear speckle marker SC35-pi staining. (a) Wild-type *ALKBH5-FLAG* and its mutants *K235R* and *K235Q* were transfected into *ALKBH5* KO HeLa cells, and ALKBH5-FLAG protein levels were determined using an anti-FLAG antibody. (b, c) *ALKBH5* KO HeLa cells stably reexpressing wild-type ALKBH5 or its mutants K235R or K235Q were treated with cycloheximide (CHX) for the indicated times. The indicated proteins were analyzed (c), and the ALKBH5 protein band densities at each time point were normalized to those of β-actin and converted into percentages using 100% as the value of the zero time point (b). (d) Immunostainings of ALKBH5-FLAG with PSPC1 was detected in *ALKBH5* KO HeLa cells stably reexpressing wild-type ALKBH5 or its mutants K235R or K235Q. Scale bar: 5 μm. (e, f) Immunostainings of ALKBH5-FLAG (e) or PSCP1 (f) and SC35-pi were detected in *ALKBH5* KO HeLa cells stably reexpressing wild-type ALKBH5 or its mutants K235R or K235Q. Scale bar: 5 μm. Source data are provided as a Source Data file.

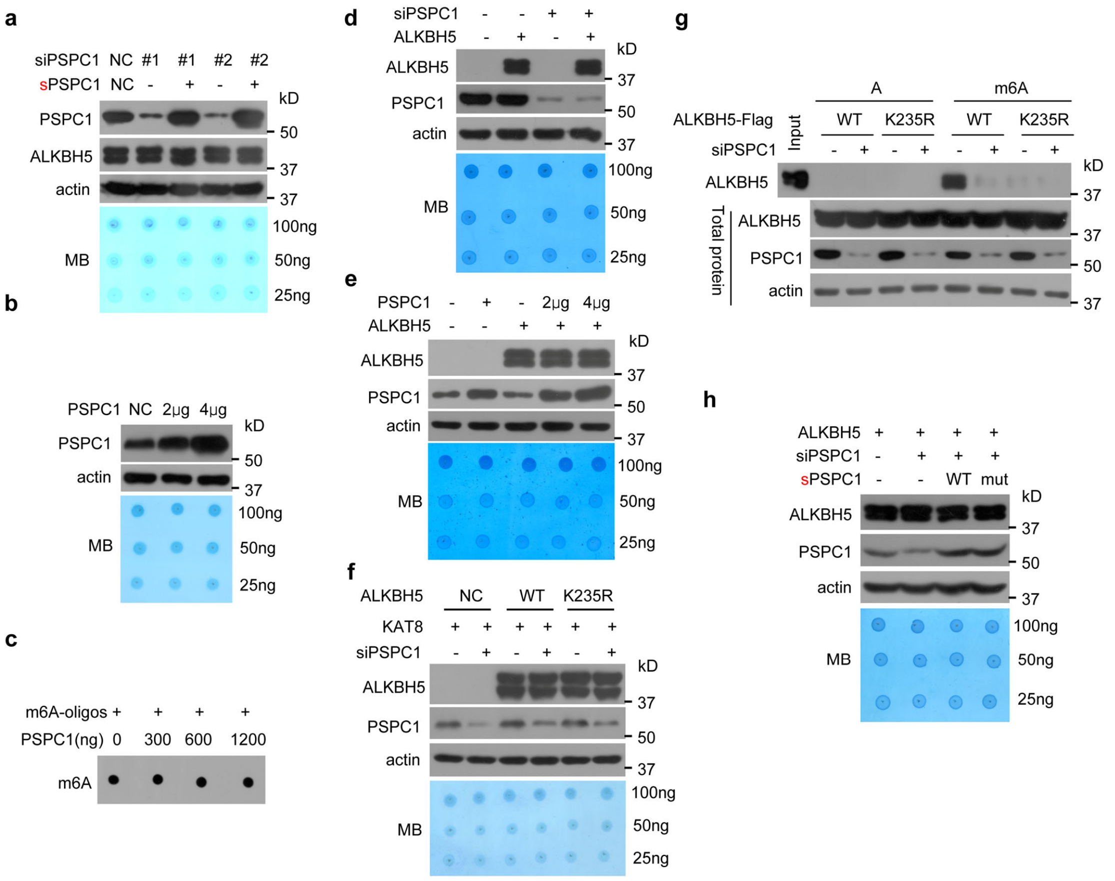


**Supplementary Figure 7.** PSCP1 itself does not change the RNA $m^6A$ level, KD of *PSPC1* destroyed the binding of ALKBH5 to RNA $m^6A$ and MB staining of the loaded RNA in Figure 7 as a loading control. (a) The indicated proteins were detected and the loaded RNA was stained with MB in Figure 7a. (b) The indicated proteins were detected and the loaded RNA was stained with MB in Figure 7b. (c) The indicated dose of immunopurified PSPC1, which was from *ALKBH5* KO cells, was incubated with $m^6A$ RNA oligos, and the $m^6A$ level was determined. (d) The indicated proteins were detected and the loaded RNA was stained with MB in Figure 7d. (e) The indicated proteins were detected and the loaded RNA was stained with MB in Figure 7e. (f) The indicated proteins were detected and the loaded RNA was stained with MB in Figure 7h. (g) *A*nti-*PSPC1* siRNA was transfected into *ALKBH5* KO HeLa cells stably reexpressing wild-type ALKBH5-FLAG or K235R mutant, and the *in vitro* binding of wild-type ALKBH5 and K235R mutant to $m^6A$-unmethylated or methylated RNA oligos were investigated. (h) The indicated proteins were detected and the loaded RNA was stained with MB in Figure 7l. Source data are provided as a Source Data file.

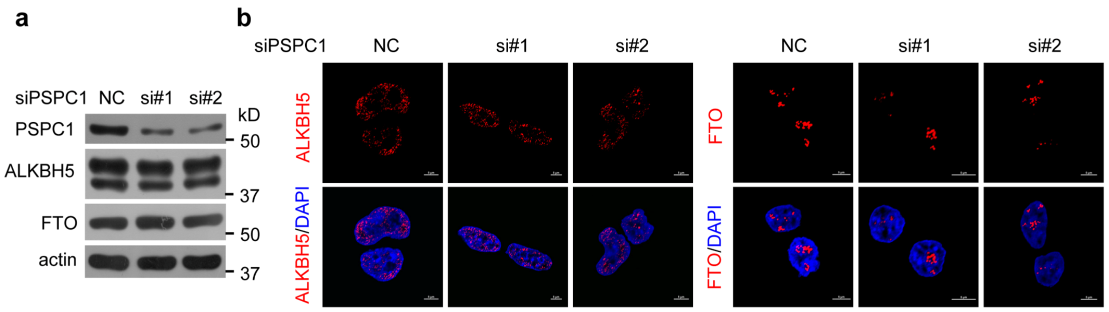


**Supplementary Figure 8.** KD of *PSPC1* did not change the level and sublocalization of the RNA $m^6A$ demethylases ALKBH5 and FTO. (a, b) Two anti-*PSPC1* siRNAs were transfected into HeLa cells, the level (a) and immunostaining (b) of ALKBH5 and FTO were determined. Source data are provided as a Source Data file.

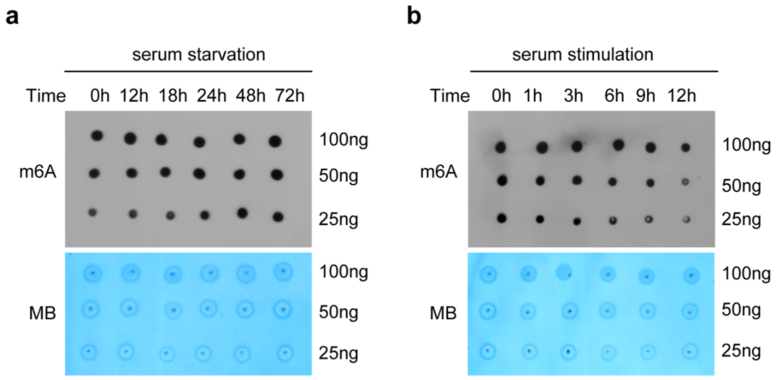


**Supplementary Figure 9.** Serum deprivation increases the cellular RNA $m^6A$ level, while serum stimulation decreases the cellular RNA $m^6A$ level. (a) HeLa cells were serum starved (0% FBS) for different times, the cellular RNA $m^6A$ level was analyzed. (b) HeLa cells were serum stimulated with the addition of 10% FBS after 2 days of serum deprivation, the cellular RNA $m^6A$ level was analyzed. Source data are provided as a Source Data file.

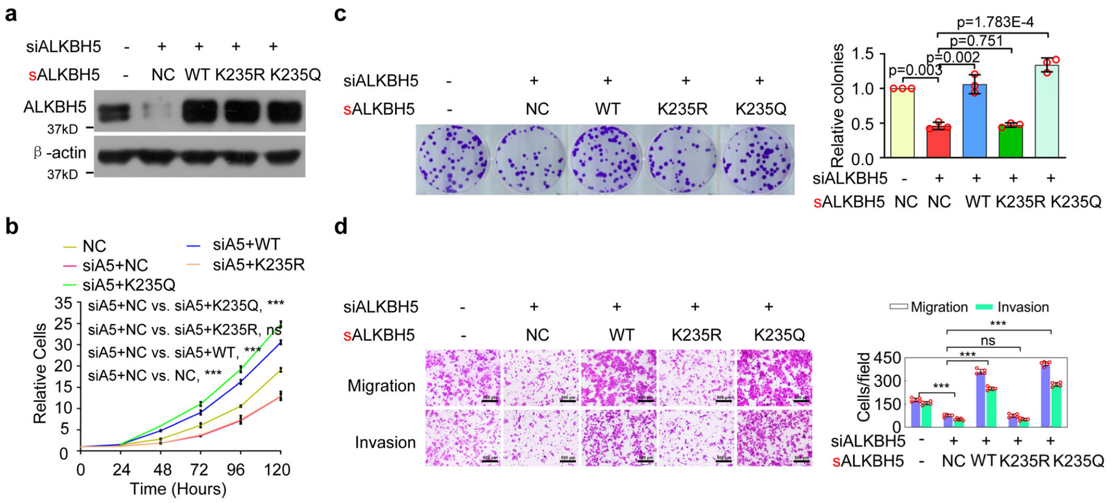


**Supplementary Figure 10.** ALKBH5 acetylation at K235 is essential for promoting cellular proliferation, colony formation, migration and invasion. The synonymously mutated wild-type s*ALKBH5* and its mutant *K235R* and *K235Q* plasmids, which were resistant to anti-*ALKBH5* siRNA, together with anti-*ALKBH5* siRNA, were cotransfected into HCT-116 cells. The ALKBH5 protein level (a), cell proliferation (b) (n=3), colony formation (c) (n=3), migration and invasion (d) (n=5) were determined. Scale bar: 500 μm. Two-tailed unpaired Student's *t*-test in (c and d), two-way ANOVA in (b). The data are represented as the means ± SD. ***p<0.001, ns indicates no significance. Source data are provided as a Source Data file.

## Supplementary Tables

**Supplementary Table 1.** The siRNA sequences used in this study.

| Gene name | siRNA No. | siRNA sequences |
|---|---|---|
| ALKBH5 | si#1 | Sense: 5'-GACUGUGCUCAGUGGAUAUTT-3'<br>Antisense: 5'-AUAUCCACUGAGCACAGUCTT-3' |
| | si#2 | sense: 5'-GCUUCAGCUCUGAGAACUATT-3'<br>antisense: 5'-UAGUUCUCAGAGCUGAAGCTT-3' |
| HDAC7 | si#1 | sense: 5'-GCACCCAGCAAACCUUCUATT-3'<br>antisense: 5'-UAGAAGGUUUGCUGGGUGCTT-3' |
| | si#2 | sense: 5'-GCACCCAGCAAACCUUCUATT-3'<br>antisense: 5'-UAGAAGGUUUGCUGGGUGCTT-3' |
| KAT8 | si#1 | sense: 5'-GCCUGGUAUUUCUCACCAUTT-3'<br>antisense: 5'-AUGGUGAGAAAUACCAGGCTT-3' |
| | si#2 | sense: 5'-GGAAAGAGAUCUACCGCAATT-3'<br>antisense: 5'-UUGCGGUAGAUCUCUUUCCTT-3' |
| PSPC1 | si#1 | sense: 5'-GCACGAAAGGCUCUGGAAATT-3'<br>antisense: 5'-UUUCCAGAGCCUUUCGUGCTT-3' |
| | si#2 | sense: 5'-GCUAAUGAGGCAAGAUCUATT-3'<br>antisense: 5'-UAGAUCUUGCCUCAUUAGCTT-3' |
| NC | | sense: 5'-UUCUCCGAACGUGUCACG-3'<br>antisense: 5'-ACGUGACACGUUCGGAGAATT-3' |
