# Supplementary material for: K235 acetylation couples with PSPC1 to regulate the m6A demethylation activity of ALKBH5 and tumorigenesis
Source: Nat Commun. 2023 Jun 27;14:3815. doi: 10.1038/s41467-023-39414-4 (PMC10300122; doi:10.1038/s41467-023-39414-4)
Supplement: Supplementary file 2 — Reporting Summary [file 41467_2023_39414_MOESM2_ESM.pdf]

nature portfolio

Corresponding author(s): Guang-Rong Yan

Last updated by author(s): Jan 10, 2023

Reporting Summary

Nature Portfolio wishes to improve the reproducibility of the work that we publish. This form provides structure for consistency and transparency in reporting. For further information on Nature Portfolio policies, see our Editorial Policies and the Editorial Policy Checklist.

Statistics

For all statistical analyses, confirm that the following items are present in the figure legend, table legend, main text, or Methods section.

n/a	Confirmed	
☐	☒	The exact sample size (*n*) for each experimental group/condition, given as a discrete number and unit of measurement
☐	☒	A statement on whether measurements were taken from distinct samples or whether the same sample was measured repeatedly
☐	☒	The statistical test(s) used AND whether they are one- or two-sided<br>*Only common tests should be described solely by name; describe more complex techniques in the Methods section.*
☒	☐	A description of all covariates tested
☒	☐	A description of any assumptions or corrections, such as tests of normality and adjustment for multiple comparisons
☐	☒	A full description of the statistical parameters including central tendency (e.g. means) or other basic estimates (e.g. regression coefficient) AND variation (e.g. standard deviation) or associated estimates of uncertainty (e.g. confidence intervals)
☐	☒	For null hypothesis testing, the test statistic (e.g. *F*, *t*, *r*) with confidence intervals, effect sizes, degrees of freedom and *P* value noted<br>*Give P values as exact values whenever suitable.*
☒	☐	For Bayesian analysis, information on the choice of priors and Markov chain Monte Carlo settings
☒	☐	For hierarchical and complex designs, identification of the appropriate level for tests and full reporting of outcomes
☒	☐	Estimates of effect sizes (e.g. Cohen's *d*, Pearson's *r*), indicating how they were calculated

Our web collection on statistics for biologists contains articles on many of the points above.

Software and code

Policy information about availability of computer code

Data collection	PEAKS Studio 8.5, Mascot (v2.3.02) , bowtie, R package exomePeak, HOMER, IGV (http://www.igv.org/)
Data analysis	Prism 8, SPSS 16.0

For manuscripts utilizing custom algorithms or software that are central to the research but not yet described in published literature, software must be made available to editors and reviewers. We strongly encourage code deposition in a community repository (e.g. GitHub). See the Nature Portfolio guidelines for submitting code & software for further information.

Data

Policy information about availability of data

All manuscripts must include a data availability statement. This statement should provide the following information, where applicable:

- Accession codes, unique identifiers, or web links for publicly available datasets
- A description of any restrictions on data availability
- For clinical datasets or third party data, please ensure that the statement adheres to our policy

The m6A-seq data of K235 acetylation-mediated m6A profiles have been deposited into the Gene Expression Omnibus (GEO) under accession number GSE142203 (https://www.ncbi.nlm.nih.gov/geo/query/acc.cgi?acc=GSE142203). The m6A-seq data of ALKBH5- and PSPC1-mediated m6A profiles have been deposited in the Genome Sequence Archive (GSA) of the BIG Data Center, Beijing Institute of Genomics (BIG, http://gsa.big.ac.cn) under accession number HRA000565 (https://bigd.big.ac.cn/gsa-human/browse/HRA000565). The mass spectrometry proteomics data on the identification of ALKBH5 acetylation and K235-acetylated ALKBH5-

interaction partners have been deposited in the ProteomeXchange Consortium via the iProX partner repository34 with the dataset identifiers PXD020070 and PXD020071, respectively. The human genome database (Version GRCh38.p12) was freely downloaded from Ensemble (www.ensembl.org). All uncropped blots are provided as a source data file. All other relevant data are available from the corresponding author (G.-R.Y.) on reasonable request.

Human research participants

Policy information about studies involving human research participants and Sex and Gender in Research.

Reporting on sex and gender	Findings do not apply to only one sex. Sex was not considered in the study design. K235 acetylation of ALKBH5 impacts on both women and men.
Population characteristics	A cohort of 5 liver cancer patients was composed of 4 males and 1 female (age from 29 to 54 years old). A cohort of 5 gastric cancer patients was composed of 3 males and 2 females (age from 43 to 72 years old).
Recruitment	Patients are randomly recruited. No self-section bias or other biases are present. These cases were selected based on a clear pathological diagnosis, and the patients were not preoperatively treated with anticancer agents.
Ethics oversight	The collection of these samples was approved by the Internal Review and Ethics Boards at the Third Affiliated Hospital of Guangzhou Medicine University. Informed consent was obtained from each patient.

Note that full information on the approval of the study protocol must also be provided in the manuscript.

Field-specific reporting

Please select the one below that is the best fit for your research. If you are not sure, read the appropriate sections before making your selection.

☒ Life sciences ☐ Behavioural & social sciences ☐ Ecological, evolutionary & environmental sciences

For a reference copy of the document with all sections, see nature.com/documents/nr-reporting-summary-flat.pdf

Life sciences study design

All studies must disclose on these points even when the disclosure is negative.

Sample size	No sample size calculation was performed. Sample size was determined from similar experiments in the literature. Sample size and the number of independent experiments are clearly stated in the figure legend, in the main text section, or in the Methods section. Three to more independent results were used to for statistical analysis. If less, statistics analysis was not performed.
Data exclusions	No data were excluded.
Replication	Experiments in the article were reliably reproduced. Detailed information on replicates was available in the figure legends.
Randomization	In mouse experiments, all mice were randomly assigned to the experimental group prior to initiation of treatment. For the remaining studies, experiments were performed in cell lines, and randomization was therefore not appropriate, but the treated groups were attributed randomly.
Blinding	Data acquisition and/or analysis in the studies, including animal experiments, was conducted in a blinded manner.

Reporting for specific materials, systems and methods

We require information from authors about some types of materials, experimental systems and methods used in many studies. Here, indicate whether each material, system or method listed is relevant to your study. If you are not sure if a list item applies to your research, read the appropriate section before selecting a response.

Materials & experimental systems

n/a	Involved in the study	
☐	☒	Antibodies
☐	☒	Eukaryotic cell lines
☒	☐	Palaeontology and archaeology
☐	☒	Animals and other organisms
☒	☐	Clinical data
☒	☐	Dual use research of concern

Methods

n/a	Involved in the study	
☒	☐	ChIP-seq
☒	☐	Flow cytometry
☒	☐	MRI-based neuroimaging

Antibodies

Antibodies used	Anti-Ac-K235 (developed in our lab, 1:500), acetylated lysine (Pan-Ac) (1:1000, 9814, CST, RRID:AB_10544700), ALKBH5 (703570, Thermo Fisher Scientific, RRID: AB_2762417, 1:1000), FLAG (M185-3L, MBL, RRID: AB_11123930, 1:2000), HA (561, MBL, RRID: AB_591839, 1:2000), V5 (66007-1-Ig, Proteintech, RRID: AB_2734694, 1:1000), KAT8 (ab200660, Abcam, 1:1000), HDAC7 (33418, CST, RRID: AB_2716756, 1:1000), PSPC1 (16714-1-AP, Proteintech, RRID: AB_2878302, 1:1000), m6A (for dot blotting, ABE572, Merck Millipore, 1:1000), m6A (for m6A-seq, 202003, Synaptic systems, PRID: AB_2279214, 1:500), GST (2625S, CST, RRID: AB_490796, 1:1000), NONO (11058-1-AP, Proteintech, RRID: AB_2152167， 1:1000), SFPQ (15585-1-AP, Proteintech, RRID: AB_10697653, 1:1000), FOXM1 (13147-1-AP, Proteintech, RRID:AB_2106213, 1:1000), SMAD7 (25840-1-AP, Proteintech, RRID:AB_2848137, 1:1000), TACC3 (25697-1-AP, Proteintech, RRID:AB_2880199, 1:1000), c-myc (13987S, CST, RRID:AB_2631168, 1:1000) and β-actin (60008-1-Ig, Proteintech, RRID: AB_2289225, 1:5000).
Validation	The anti-Ac-K235 antibody developed in our laboratory was validated by our group. The related data are provided in Supplementary Figure 2. All of the commercially available antibodies used in this study were validated for the use in human specimens by the manufacturers and for the respective methods used in this manuscript (see home pages of respective manufacturers using catalogue numbers provided above).

Eukaryotic cell lines

Policy information about cell lines and Sex and Gender in Research

Cell line source(s)	HeLa, HEK293T and HCT-116 cell lines were from the American Type Culture Collection (ATCC, USA) and cultured under standard conditions.
Authentication	The cell lines were authenticated by ATCC via STR profiling.
Mycoplasma contamination	Cells were regularly monitored for mycoplasma contamination. All cell lines tested negative for mycoplasma contamination.
Commonly misidentified lines (See ICLAC register)	No commonly misidentified cell lines were used.

Animals and other research organisms

Policy information about studies involving animals; ARRIVE guidelines recommended for reporting animal research, and Sex and Gender in Research

Laboratory animals	Female BALB/c nude mice (3-4 weeks old) were purchased from Guangdong Medical Animal Experiment Center (Guangzhou, China). The mice used in these experiments were bred and maintained under defined conditions (at room temperature (20-26 °C) and 40-70% humidity with a 12 h light/dark cycle and access to food and water ad libitum) at the Guangdong Medical Animal Experiment Center (SPF-grade facility).
Wild animals	No wild animals were involved in this study.
Reporting on sex	Findings do not apply to only one sex. Sex was not considered in the study design. K235 acetylation of ALKBH5 impacts on both women and men.
Field-collected samples	The study did not involves samples collected from the field.
Ethics oversight	The animal experiments were approved by the Laboratory Animal Ethics Committee of the Third Affiliated Hospital of Guangzhou Medicine University and Guangdong Medical Animal Experiment Center and conformed to the legal mandates and national guidelines for the care and maintenance of laboratory animals. The maximal tumor size permitted by this ethics committee should not exceed 20 mm in any one dimension. In this study, the maximal tumor size was not exceeded 20 mm in any one dimension.

Note that full information on the approval of the study protocol must also be provided in the manuscript.
